# A meta-analysis study of the robustness and universality of gut microbiome-metabolome associations

**DOI:** 10.1186/s40168-021-01149-z

**Published:** 2021-10-12

**Authors:** Efrat Muller, Yadid M. Algavi, Elhanan Borenstein

**Affiliations:** 1 The Blavatnik School of Computer Science, Tel Aviv University, Tel Aviv, Israel; 2 Sackler Faculty of Medicine, Tel Aviv University, Tel Aviv, Israel; 3 Santa Fe Institute, Santa Fe, NM USA

**Keywords:** Gut microbiome, Metagenomics, Metabolomics, Multi-omic, Meta-analysis, Machine learning

## Abstract

**Background:**

Microbiome-metabolome studies of the human gut have been gaining popularity in recent years, mostly due to accumulating evidence of the interplay between gut microbes, metabolites, and host health. Statistical and machine learning-based methods have been widely applied to analyze such paired microbiome-metabolome data, in the hope of identifying metabolites that are governed by the composition of the microbiome. Such metabolites can be likely modulated by microbiome-based interventions, offering a route for promoting gut metabolic health. Yet, to date, it remains unclear whether findings of microbially associated metabolites in any single study carry over to other studies or cohorts, and how robust and universal are microbiome-metabolites links.

**Results:**

In this study, we addressed this challenge by performing a comprehensive meta-analysis to identify human gut metabolites that can be predicted based on the composition of the gut microbiome across multiple studies. We term such metabolites “robustly well-predicted”. To this end, we processed data from 1733 samples from 10 independent human gut microbiome-metabolome studies, focusing initially on healthy subjects, and implemented a machine learning pipeline to predict metabolite levels in each dataset based on the composition of the microbiome. Comparing the predictability of each metabolite across datasets, we found 97 robustly well-predicted metabolites. These include metabolites involved in important microbial pathways such as bile acid transformations and polyamines metabolism. Importantly, however, other metabolites exhibited large variation in predictability across datasets, suggesting a cohort- or study-specific relationship between the microbiome and the metabolite. Comparing taxonomic contributors to different models, we found that some robustly well-predicted metabolites were predicted by markedly different sets of taxa across datasets, suggesting that some microbially associated metabolites may be governed by different members of the microbiome in different cohorts. We finally examined whether models trained on a control group of a given study successfully predicted the metabolite’s level in the disease group of the same study, identifying several metabolites where the model was not transferable, indicating a shift in microbial metabolism in disease-associated dysbiosis.

**Conclusions:**

Combined, our findings provide a better understanding of the link between the microbiome and metabolites and allow researchers to put identified microbially associated metabolites within the context of other studies.

**Video abstract**

**Supplementary Information:**

The online version contains supplementary material available at 10.1186/s40168-021-01149-z.

## Background

The microbial communities that reside in the human gut wield a multitude of activities with pervasive effect on human health and disease. Among these activities, perhaps the most important and clinically relevant one is the microbiota’s metabolic activity. Indeed, metabolites of microbial origin or metabolites that undergo microbial transformation have been implicated in various host processes, including immune system development and activity, host metabolism, and even brain function [1–4]. Several recent studies have also established a causal link between microbially produced metabolites and various medical conditions (or benefits). One well-known example is that of trimethylamine *N*-oxide (TMAO), a product of microbial metabolism of nutrients found in eggs and red meat, which accelerates atherosclerotic cardiovascular diseases [5, 6]. Other examples include imidazole propionate, a metabolite over-produced by Type 2 Diabetes-associated bacteria, which was found to impair glucose tolerance [7]*, Akkermansia muciniphila*-associated nicotinamide, which was shown to improve motor symptoms in a mouse model of ALS [8], and microbially produced short-chain fatty acids (SCFAs), which have a protective role against intestinal inflammation [9]. Other microbial metabolites protect from influenza [10] and even improve mental quality of life [11]. Moreover, microbiome-associated metabolites such as the organic acid taurine or some SCFAs have been studied as potential novel therapeutic agents, intended to “correct” negative effects of microbial dysbiosis [12].

This appreciation for the metabolic role of the gut microbiome in maintaining host health or promoting disease calls for a better understanding of which gut metabolites are governed by the microbiota, and specifically by which members of the microbiota. To address this challenge, in vitro culturing of common gut strains [13, 14], in vivo experiments comparing germ-free mice to humanized mice [15], as well as bioinformatic analyses of bacterial genomes [16], have all been applied in an attempt to map the metabolic potential of the human gut microbiota. However, additional factors shaping the human gut metabolome, including host genetics, diet, medications, as well as other exogenous factors, render an extremely complex system in which the exact role of the microbiota remains challenging to untangle [3, 17].

One promising approach for systematically evaluating microbe-metabolite links in the human gut relies on integrative data analysis of paired microbiome and metabolome profiles [18–32]. Such studies, referred to here as ‘microbiome-metabolome’ studies, typically apply high-throughput sequencing and metabolomics technologies to a set of fecal samples from a cohort of interest, and then utilize various statistical and computational methods to identify potential links between the obtained microbiome and metabolome profiles. Such analyses commonly involve correlation- and linear regression-based methods, aiming to estimate relations between specific metabolites and specific microbes or between specific metabolites and the entire microbial community. More recently, classic machine learning and deep learning models have also been applied to predict metabolite levels based on microbiome data and to highlight the main taxa associated with each metabolite [33–35]. MelonnPan, for example, uses an elastic net model to predict metabolite levels based on functional profiles of microbiome samples, and was shown to well-predict 107 out of 466 identified metabolites, including sphingolipids, fatty acids, and B-group vitamins [33].

Such microbiome-metabolite associations reported in individual studies of the human gut, however, raise several questions. First, it remains unclear what do such associations mean biologically, and whether or not they reflect underlying mechanisms. In fact, our lab has recently shown that microbe-metabolite correlations have extremely high false positive rates in predicting mechanistic links in simulated data [36]. Second, in the context of case-control studies, it is often unclear how the association is related to the phenotype/disease that is under study and how to interpret findings of associations that exist in one study group but not the other vs those that exist in both groups. Third, it is unclear how generalizable are such associations, given that they were identified in a specific cohort, often using a specific computational method and a specific metagenomics/metabolomics processing protocol. Put differently, it is often not known how replicable are reported associations in other human gut datasets of varying geographies, ages, sample processing protocols, and profiling platforms [37, 38]. Moreover, even when analyzing large cohorts, the transferability of microbiome-based metabolite predictions to new cohorts is not guaranteed, as recently demonstrated on serum metabolites [35].

In this study, we focus primarily on the third question above and attempt to characterize the landscape of gut metabolites that are consistently well-predicted by the gut microbiome, as captured in fecal microbiome-metabolome datasets. We hypothesize that some of the reported microbiome-associated metabolites are biologically meaningful, representing metabolites that are universally governed by the microbiome across different cohorts and biological backgrounds, and are robust to the variation in the technical settings used in each study. We accordingly aim to identify a set of gut *microbiome*-metabolite associations, consistent across multiple cohorts and settings, and explore the specific genera contributing to the predictability of each such metabolite. To this end, we obtained multiple paired fecal microbiome-metabolome datasets and focused on the healthy individuals in each dataset. For each dataset, we then trained and evaluated a machine learning model per metabolite, aiming to predict the metabolite level in each sample based on microbiota composition. We examined which metabolites were consistently well-predicted across multiple datasets, using rigorous statistical methods inspired by traditional meta-analysis techniques to define “robustly well-predicted” metabolites. Lastly, we analyze which genera contribute most to the predictability of each robustly well-predicted metabolite, and whether these too are consistent across datasets and in healthy vs. disease cohorts. We believe that this cross-study perspective on suspected microbial-governed metabolites in the human gut is crucial for prioritizing further research hypotheses and for enhancing interpretations of future microbiome-metabolome studies by providing relevant context.

## Results

### A unified human fecal microbiome-metabolome multi-study dataset collection

**Fig. 1:**
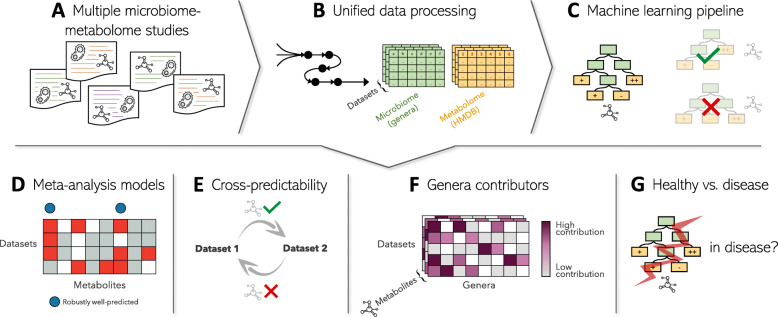
Analysis scheme. **A** We collected data from multiple studies that included both microbiome and metabolome profiles from human fecal samples. Data from case-control studies were split into two datasets: “healthy” and “disease”. **B** We implemented a processing pipeline for both the microbiome and the metabolome data. For the microbiome, we collapsed MetaPhlAn-based or 16S rRNA gene-based profiles into genus-level profiles with unified names. For the metabolome, we retained only metabolites for which HMDB-IDs were identified, imputed missing values, and scaled log values to zero-mean unit-variance (see “Methods” section). **C** For each metabolite in each dataset, we trained a random forest regression model (see “Methods” section). Models were only trained for metabolites that appeared in 3 or more datasets. We identified the well-predicted metabolites in each dataset, i.e., metabolites that can be successfully predicted by genus-level profiles of the gut microbiota (Spearman’s *ρ* > 0.3 and FDR-corrected *P* value < 0.1 on out-of-fold predictions). **D** We next applied meta-analysis random-effects models to compare metabolite predictability results across datasets and identify metabolites which are consistently well-predicted by the microbiota’s composition. **E** To further evaluate whether robustly well-predicted metabolites also demonstrate similar dynamics in relation to the microbiome across datasets, we analyzed how well metabolite models trained on one dataset perform on another dataset, using only shared genera features. **F** We additionally identified the main genera contributors to the model and again compared contributors across datasets to evaluate the similarity between models trained on different datasets for the same metabolite, and identify consistent contributors. **G** Lastly, we identified metabolites for which genera contributors change in disease

**Fig. 2:**
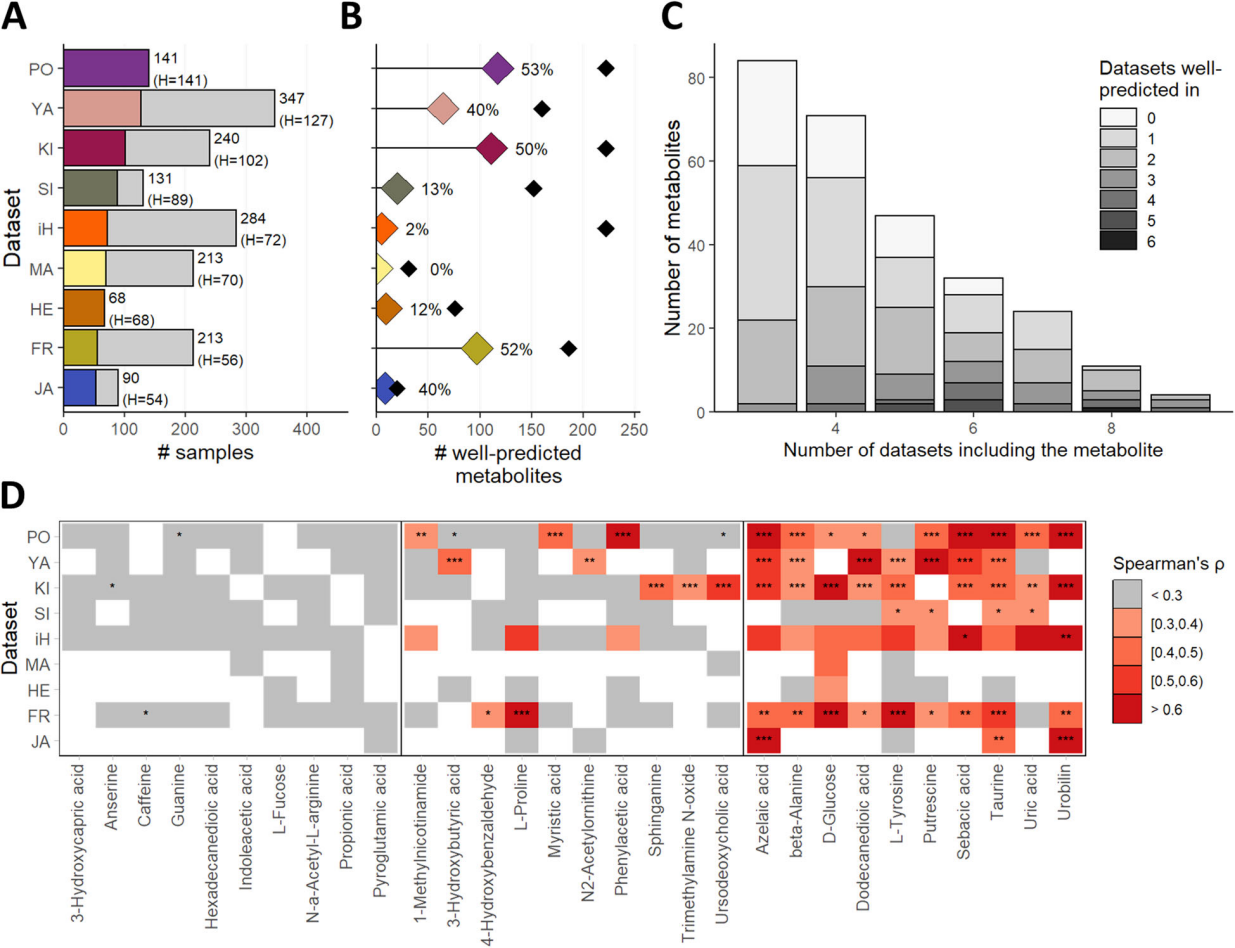
Identification of metabolites well-predicted by the microbiome using machine learning. **A** Number of samples per dataset. Colored portions of each bar represent samples from the healthy/control study group. **B** The number of well-predicted metabolites in each dataset. Black diamonds represent the total number of metabolites analyzed per dataset (with the percentage labels indicating the percent of analyzed metabolites that were well-predicted in each dataset). **C** The number of datasets each metabolite was well-predicted in, stratified by the number of datasets each metabolite appeared in. **D** Examples of predictability results for 30 metabolites. Each heatmap row represents a dataset and each column denotes a specific metabolite. Cell colors and labels represent predictability levels (Spearman’s ρ on out-of-fold predictions and FDR-corrected *P* values: *FDR < 0.05; **FDR < 0.01; ***FDR < 0.001). White cells indicate that the metabolite was not available in that dataset. The examples presented here include metabolites never well-predicted (leftmost panel), well-predicted in only one dataset (center panel), and well-predicted in several datasets (rightmost panel)

We collected and processed data from 10 different human gut microbiome-metabolome studies, totaling 779 samples from 629 individuals in ‘healthy’ (‘control’) groups and 954 samples from 729 individuals in 7 ‘disease’ (‘case’) groups (Table 1, Fig. 1A, Additional file 1: Table S1). Notably, these studies spanned various ages, geographies, health conditions, metagenomics/metabolomics platforms, and 16S rRNA gene hypervariable regions, all of which are expected to introduce heterogeneity between datasets, as demonstrated in previous microbiome and metabolome meta-analysis studies in various fields [44–46]. Importantly, in case-control studies, we treated healthy and disease subgroups separately (considering only study groups with ≥ 40 samples) to avoid the confounding impact of the disease state on both the microbiome and metabolome compositions. Moreover, to first focus on robustly well-predicted metabolites in general population-like cohorts, in our analysis below we initially considered *only* the healthy datasets (Fig. 2A).

**Table 1:** Studies included in the main analysis (additional details provided in Additional file 1: Table S1)

Dataset ID [abbreviation] ^1^	Reference	# samples – control^2^	# samples – cases (disease/condition)^2^	Microbiome data description	Metabolome data description	# metabolites^3^
KIM_ADENOMAS [KI]	[27]	102	138 (Colorectal adenomas and CRC)	16S, V3-V5	Untargeted, MS	410
YACHIDA_CRC [YA]	[39]	127	220 (CRC)	WGSS	Targeted, MS	387
FRANZOSA_IBD [FR]	[22]	56	157 (IBD)	WGSS	Untargeted, MS	294
HE_INFANTS [HE]	[40]	68	–	16S, V4	Targeted, NMR	113
iHMP_IBD [iH]	[19]	72 [26]	212 [79] (IBD)	WGSS	Untargeted, MS	504
JACOBS_IBD_RELATIVES [JA]	[21]	54	–^5^	16S, V4	Untargeted, MS	45
MARS_IBS [MA]	[41]	70 [24]	143 [51] (IBS)	WGSS	Targeted, NMR, and MS	40
POYET_BIO_ML [PO]	[26]	141 [83]	–	16S^4^, V4	Untargeted, MS	313
SINHA_CRC [SI]	[42]	89	42 (CRC)	16S, V3-V4	Untargeted, MS	351
ERAWIJANTARI_GC [ER]	[43]	–^5^	42 (History of gastrectomy for GC)	WGSS	Untargeted, MS	342

^1^The “Dataset ID” is formatted as follows: <First author/cohort name>_<Short cohort description>. The 2 letter abbreviations are used for plotting purposes

^2^Sample numbers refer only to samples included in the analysis herein. In cases of temporal datasets with multiple samples per individual, the number of individuals is noted in square brackets

^3^Number of HMDB-annotated metabolites. See “Methods” section

^4^WGSS data was also available but not used in the analysis herein

^5^This study group was not used

*CRC* colorectal cancer, *MS* mass spectrometry, *16S* 16S rRNA gene sequencing, *WGSS* whole genome shotgun sequencing, *IBD* inflammatory bowel disease, *IBS* irritable bowel syndrome, *GC* gastric cancer

Microbiome data was processed to obtain genus-level profiles, providing more comparable taxonomic profiles across 16S rRNA gene sequencing and whole genome shotgun sequencing (WGSS) datasets at the expense of sensitivity and resolution (Fig. 1B), as done in several recent microbiome-related meta-analysis studies [44, 47]. Specifically, when possible, 16S rRNA gene sequencing raw data were re-processed using QIIME2 [48] to obtain genus-level relative abundances and MetaPhlAn2 tables were collapsed to genus-level profiles (see full details in Additional file 1: Table S2). Metabolite identifications were unified by converting the identifications given in each dataset to the Human Metabolome Database (HMDB) metabolite identifiers [49]. Further processing details, statistics, and study-specific required adjustments are detailed in the “Methods” section and in Additional file 1: Tables S2–S4.

Clearly, the set of detected features in both microbiome and metabolome profiles (i.e., genera and metabolites, respectively) are expected to differ between studies due to both technical [2, 50, 51] and biological factors [20, 52, 53]. This limited overlap across datasets hinders in and of itself our ability to carry microbiome-metabolite links over different studies, and is discussed further in the “Discussion” section below. Here, in order to detect consistently well-predicted metabolites, we first examined which gut metabolites are shared between datasets, limiting our analysis to those that appear in 3 or more datasets. Out of 951 unique, non-rare, HMDB compound IDs found across all datasets, 273 (29%) were shared among 3 or more datasets (Additional file 3: Figure S1A). We additionally examined which genera are shared between datasets, to facilitate specific analysis of contributors to well-predicted metabolites. Out of 85 unique, none-rare genera found across all datasets, 55 (65%) were shared between 3 or more datasets (Additional file 3: Figure S1B). Pairwise comparisons between studies in terms of overlap in microbial and metabolic features further demonstrate the large variability and often limited overlap (Additional file 3: Figure S1C-D).

### Predictability of metabolite levels based on microbiome data

We implemented a machine learning pipeline to estimate how well genus-level profiles can predict metabolite levels in each dataset and for each metabolite (Fig. 1C). Specifically, for each HMDB-annotated metabolite that appeared in 3 or more datasets, we trained a random forest regression model to predict metabolite levels based on genera relative abundances. Alternative pipelines with either a different machine learning model or a different hyperparameter tuning process were examined as well (see Additional file 2: Supplementary Note 1). We evaluated each model’s performance using leave-one-out cross validation by calculating the Spearman’s correlation coefficient, *ρ*, between actual vs predicted left out metabolite levels. Spearman’s correlation *P* value was also recorded, and FDR-correction was applied to all metabolite-models in each dataset (see “Methods” section). Metabolites with a predictability of *ρ* > 0.3 and an FDR < 0.1 were referred to as ‘well-predicted’ metabolites.

Overall, 1255 metabolite predictor models (i.e., for a specific metabolite in a specific dataset) were trained, covering 273 unique metabolites in 9 healthy datasets. Of these, 418 models were able to successfully predict the metabolite level (with *ρ* > 0.3 and FDR < 0.1), and accordingly defined as well-predicted. In each individual dataset, 0–53% of the analyzed metabolites were well-predicted (Fig. 2B). Of the 273 unique metabolites, 219, 125, and 49 metabolites were well-predicted in at least 1, 2, or 3 datasets, respectively, while 54 were never well-predicted (Fig. 2C, D). Full predictability results for each metabolite in each dataset are provided in Additional file 1: Table S5.

We further validated that these predictability results cannot be attributed to statistical noise or other artifacts in the data by comparing the fraction of well-predicted metabolites in each dataset to the fraction of well-predicated metabolites obtained in a shuffled dataset (see “Methods” section). We found that in such shuffled datasets only 0.3% of the metabolites in each dataset were well-predicted on average, compared to 29.1% well-predicted metabolites in the real data. As an additional independent validation for our machine-learning pipeline, we confirmed that our set of well-predicted metabolites in one specific dataset exhibits marked overlap with the set of predictable metabolites found by another study that analyzed this same dataset (see Additional file 2: Supplementary Note 2 and Additional file 3: Figure S2A).

### Robustness of metabolite predictability

**Fig. 3:**
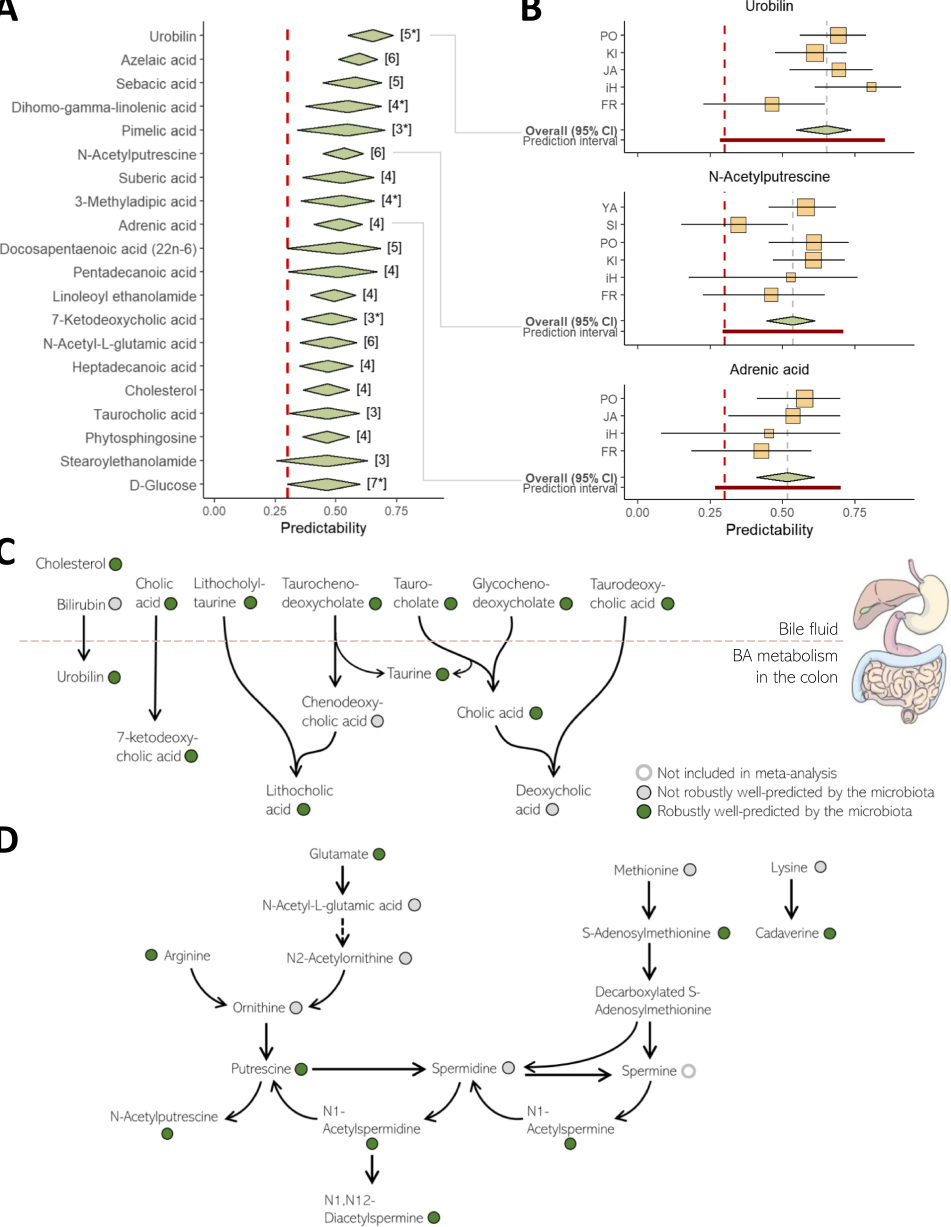
Robustly well-predicted metabolites. **A** Top 20 robustly well-predicted metabolites. Diamonds’ centers represent the random-effects model’s estimated mean effect size (mean predictability) and diamonds’ widths represent the mean’s 95% confidence interval. The numbers in square brackets represent the number of datasets in which the metabolite was available. A star beside the number of datasets indicates that in one or more of the datasets, this metabolite was annotated with low confidence (see “Methods” section). The red dashed line represents a Spearman’s correlation of 0.3, which we defined as the threshold for a successful predictive model. **B** Forest plots of 3 robustly well-predicted metabolites. Yellow boxes represent predictability estimates from each dataset, with box sizes proportional to the number of samples and horizontal lines denoting the 95% confidence interval for each estimate. The diamonds and red dashed line are as above. Prediction intervals (dark red interval) further indicate the predicted range of the effect in 95% of unobserved studies [54]. **C** An illustration of a part of the bile metabolism pathway (including primary bile acids, secondary bile acids, and other bile components). Colored circles indicate whether the metabolite was robustly well-predicted by the gut microbiome (green), not robustly well-predicted (gray), or not included in the analysis (white). **D** An illustration of parts of the polyamines metabolic pathways. Color coding is as in panel **C**.

While the results reported above already demonstrate intriguing variation in predictability and highlight several metabolites that appear to be well-predicted across multiple datasets, we next applied a more rigorous statistical approach for synthesizing and combining the predictability results obtained for each dataset independently and for quantifying how *robustly* well-predicted is each metabolite. Specifically, we used random-effects models (REM)—a common meta-analysis statistical framework for integrating effect sizes from multiple studies with different sample sizes and study designs, and for calculating an estimate for the mean effect across studies and its associated *P* value [54] (see “Methods” section). We applied this framework to the predictability results obtained above for each metabolite, using the level of predictability (scored by Spearman’s *ρ*) as the measured effect size in each dataset. The resulting REM for each metabolite thus provides an estimation of the mean predictability of that metabolite across datasets, with an associated *P* value (that was FDR-corrected to account for multiple REMs). Finally, we defined metabolites with a REM predictability score > 0.3 and FDR < 0.1 as ‘*robustly well-predicted*’, resulting in a total of 97 robustly well-predicted metabolites (Fig. 3A, B and Additional file 1: Table S6). For comparison, only 87.7 ± 5.5 metabolites met the above threshold on average when applying our pipeline to datasets where metabolite labels were shuffled (see “Methods” section), suggesting that the number of robustly well-predicted metabolites in the original dataset is somewhat higher than expected by chance (*P* = 0.053).

Examining the resulting robustly well-predicted metabolites, we found that they span multiple metabolite classes including amino acids, carbohydrates, and bile acids, and are significantly enriched in the “fatty acids and conjugates” class (FDR-corrected *P* value: 0.026, Fisher’s exact test, Additional file 3: Figure S3A-B). Importantly, our set of robustly well-predicted metabolites included multiple metabolites that take part in clinically important pathways known to involve the gut microbiome. For example, this set highlights the microbiota’s known essential role in the transformation of primary bile acids to secondary bile acids and the metabolism of other bile components [55] (Fig. 3C). Additional pathways in which multiple metabolites were robustly well-predicted by the microbiome included trimethylamine N-oxide (TMAO) metabolism, tryptophan, and indole derivatives metabolism (Additional file 3: Figure S3C), polyamine biosynthesis (Fig. 3D), poly-unsaturated fatty acids (PUFAs), specifically omega-3 and omega-6 acids pathways (Additional file 3: Figure S3D-E), l-proline biosynthesis, sugars metabolism, and metabolites involved in the gut-brain axis, all of which are known to involve the gut microbiota. We additionally found that robustly well-predicted metabolites tend to have a larger proportion of their variance explained by the microbiome, as estimated by an independent study of the largest microbiome-metabolome dataset collected to date [20] (see Additional file 2: Supplementary Note 2 and Additional file 3: Figure S2B).

Interestingly, we also found several robustly well-predicted metabolites that are only rarely considered in the context of the gut microbiota. One such example is medium-chain fatty acids (MCFAs), including specifically caproic acid (HMDB0000535), pentadecanoic acid (HMDB0000826), and heptanoic acid (HMDB0000666). MCFAs are highly abundant components in dairy foods and have an essential physiological role as efficient cell energy sources [56]. A recent study demonstrated a significant association between dairy foods intake and the microbiota’s composition, suggesting an intriguing interaction between gut microbes and these MCFAs [57]. Another example is dicarboxylic acids, including specifically malonic acid (HMDB0000691), undecanedioic acid (HMDB0000888), pimelic acid (HMDB0000857), suberic acid (HMDB0000893), sebacic acid (HMDB0000792), azelaic acid (HMDB0000784), and dodecanedioic acid (HMDB0000623). The clinical relevance of both metabolite classes, specifically in the context of type 2 diabetes [58, 59], and their relatively unexplored relation with the gut microbiome, suggest interesting directions for future research. Additional file 2: Supplementary Note 3 details additional intriguing information concerning the set of robustly well-predicted metabolites, their biological roles, and previously reported links to the microbiome.

### Similarity and variation between metabolite models from different datasets

**Fig. 4:**
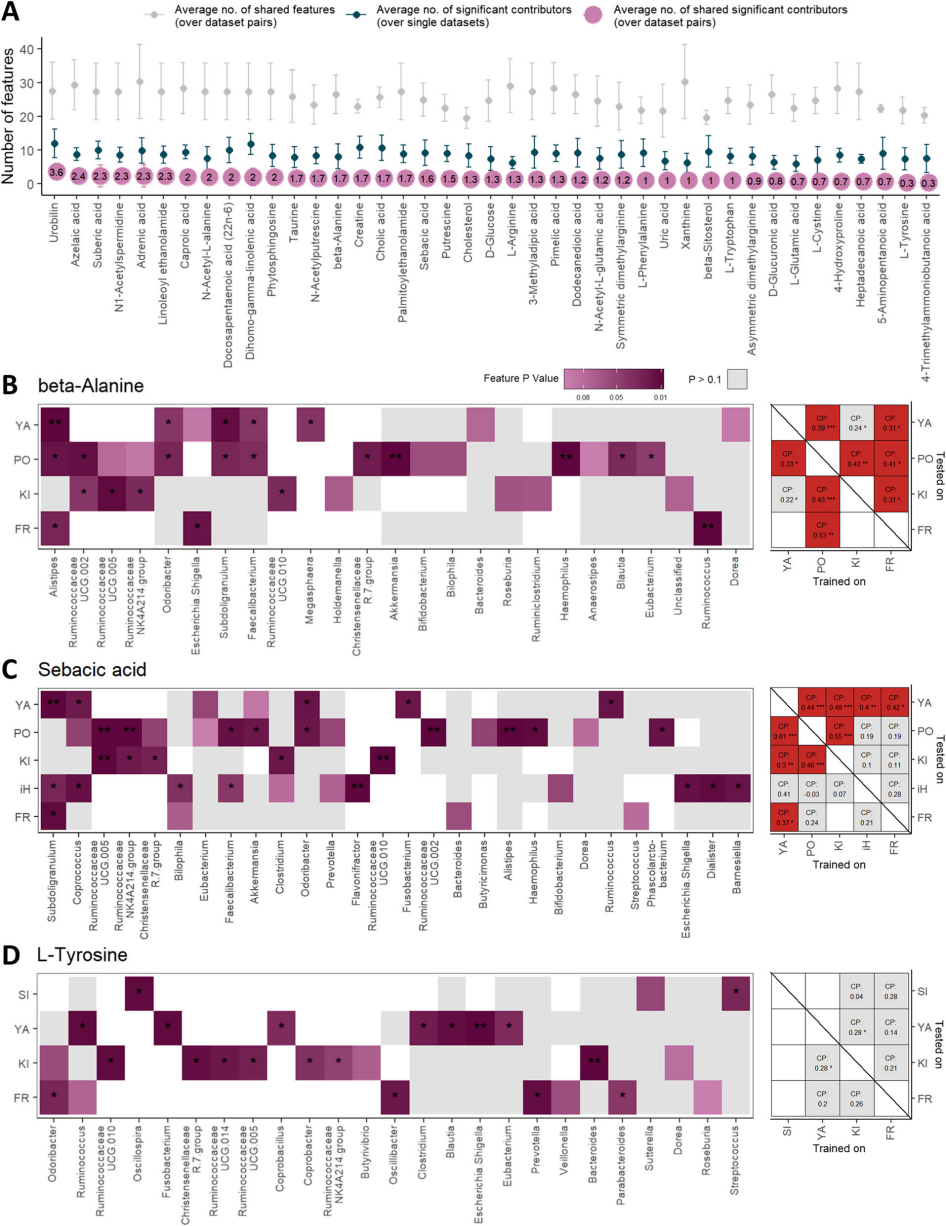
Comparisons of metabolite models between datasets. **A** An overview of pairwise model comparisons for each robustly well-predicted metabolite. For each metabolite, we compared the number of shared features (i.e., genera; grey points) and the number of shared significant contributors (purple points) for every pair of models trained on two different datasets. For each metabolite we additionally note the average number of significant contributors over all models trained for that metabolite (turquoise points). Error bars represent standard deviations. **B** A detailed comparison of models for predicting beta-alanine levels. Left panel: contributors comparison. Each row represents a dataset in which the metabolite was well-predicted by the microbiome and each column represents a genus feature (only features significantly contributing to at least one model are included). Purple-colored cells denote the significance of the specific feature in the specific model (*P* < 0.1). White cells indicate that the feature was not available in the specific dataset. Right panel: Cross-predictability analysis. Matrix columns indicate the dataset used for training and matrix rows indicate the dataset used for testing. Numbers in cells indicate Spearman’s correlation between predicted and actual metabolite levels in the test dataset. Red cells denote cases where the model was well transferred from the column-dataset to the row-dataset. **C**. **D** include similar plots as **B**, for sebacic acid and l-tyrosine metabolites, respectively. *P < 0.05; **P < 0.01; ***P < 0.001; CP cross predictability

So far, we have identified metabolites that are consistently well-predicted by the composition of the human gut microbiota across multiple datasets. We next sought out to examine whether the models trained for predicting these metabolites across the different datasets are in fact similar to one another. To this end, we first estimated the contribution of each genus feature to each model using the permutation-based approach from Altman et al. (2010) to assign a *P* value for each feature in each model (“Methods” section and Additional file 1: Table S7). We refer to genera with *P* < 0.1 as significant contributors. Comparing models trained on different datasets for the same metabolite, we observed substantial variation in the numbers of shared significant contributors (Fig. 4 and Additional file 3: Figure S4), suggesting that even robustly well-predicted metabolites may not necessarily be predicted by the same microbiota features in different datasets.

To further examine the similarity between models, we also looked at cross-predictability, i.e., how well does a metabolite model trained on one dataset predict the levels of the metabolite in samples from another dataset (considering only shared genus features; see “Methods” section). Mirroring our findings above, for some metabolites such as beta-alanine, sebacic acid, taurine, and N-acetylputrescine, models were successfully transferred between most dataset pairs (Fig. 4B, C, Additional file 3: Figure S4A-B), whereas for other metabolites, such as l-tyrosine and xanthine, models were not transferable between *any* pair of studies (Fig. 4D, Additional file 3: Figure S4C).

Importantly, model comparisons, either by comparison of shared contributors or by cross-predictability analysis, are somewhat impeded by technical variation between studies, and specifically by differences in the microbiome profiling technology used (16S rRNA gene vs. WGSS). Indeed, dataset pairs that used the same metagenomic technology have a significantly higher number of shared features in comparison with dataset pairs that used different technologies (29.6 vs. 22.3 on average; Mann-Whitney *P* value < 0.0001), which in turn affects the number of shared significant contributors (1.4 vs. 0.6, *P* value < 0.0001). Yet, we did find examples where despite a large overlap in available features, models still markedly differed in the set of detected significant contributors, as was the case, for example, for l-tyrosine and cholic acid and when comparing the models obtained for the datasets YACHIDA_CRC_HEALTHY (YA) vs FRANZOSA_IBD_HEALTHY (FR) (Fig. 4D, Additional file 3: Figure S4D).

Notably, comparing the set of significant contributors across models and datasets can further provide intriguing and valuable insights. For example, we defined genera that contributed to over 50% of the metabolite models they participated in, “consistent contributors” to that metabolite (“Methods” section; Additional file 1: Table S8), and found that often such genus-metabolite links coincide with known metabolic capacities of certain gut bacteria. Bacteroides, for example, consistently contributed to models of several bile components, whereas Alistipes consistently contributed to various polyamine models, both in agreement with experimental findings [55, 60, 61] (see Additional file 2: Supplementary Note 3 for additional examples). We also note that even though genera more abundant in the gut were more likely to significantly contribute to more metabolite models (Spearman’s correlation 0.45, *P* value < 0.0001), some genera strongly contributed to the predictability of multiple gut metabolites even though their abundance in the gut is typically low. Odoribacter, for example, a member of the Bacteroidetes phylum whose mean relative abundance in our data was 0.4%, was a significant contributor in 94 of 223 models it participated in. This genus was also a consistent contributor to models of multiple bioactive metabolites such as l-phenylalanine, pimelic acid, linoleoyl ethanolamide, and l-tryptophan. Similarly, Haemophilus (mean relative abundance in our data of 0.4%), from the Proteobacteria phylum, was a significant contributor in 49 of 126 models. Finally, we note that the average number of significant contributors per metabolite ranged from 5.6 (l-Arginine) to 10.6 (Cholic acid and Dihomo-gamma-linolenic acid), suggesting an association with multiple different genera.

### Metabolite predictability in health vs. disease

We eventually sought to determine whether various patterns identified above for the healthy datasets also hold in disease. To this end, we additionally considered the 7 disease datasets obtained from the same studies as described above (Table 1). These included 3 datasets of patients with colorectal adenomas or cancer, 2 of patients with inflammatory bowel disease (IBD), 1 of patients with a history of gastric cancer, and 1 of patients with irritable bowel syndrome (IBS), totaling 954 samples (Table 1, Additional file 1: Table S1).

We first examined whether robustly well-predicted metabolites were also consistently well-predicted in the disease datasets. We accordingly used an expansion of REMs (termed subgroup meta-analysis) that considers studies from two or more different cohort types (here: healthy and disease) and estimates both the mean effect within each subgroup of studies, as well as the between-subgroup differences (see “Methods” section). Out of 97 robustly well-predicted metabolites identified above, 81 were also present in at least 3 disease datasets, and of these 57 (70%) were also robustly well-predicted in disease datasets using the same thresholds as before (Additional file 1: Table S9, Additional file 3: Figure S5). Importantly, even though the remaining 24 metabolites did not meet our criteria, none exhibited a significantly different effect than the one observed in the healthy datasets (as estimated by the *Q* statistic), suggesting that statistical power was insufficient in these cases.

Finally, we examined whether metabolites that are well-predicted in both the healthy and disease datasets of a specific study, are well-predicted by similar models. As before, we compared models both by comparing their significant contributors and by testing how well the healthy-trained model transfers to the pertaining disease dataset. Notably, here we analyzed each case-control study independently, allowing us to eliminate potential heterogeneity between datasets, and focus on model-differences associated with the disease. Overall, we performed healthy vs. disease comparisons for 128 metabolites over 5 studies in which both healthy and disease datasets were available (covering 83 distinct metabolites). We observed that many metabolite models transferred well from healthy to disease in different studies, suggesting that microbiome-metabolite dynamics in these cases are not substantially altered in the disease state. Docosapentaenoic acid (22n-6), 12,13-DHOME, and phytosphingosine, for example, transferred well from healthy to disease in both an IBD cohort and a colorectal adenomas cohort. N-acetylputrescine, pimelic acid, taurine, and tryptamine similarly transferred well in two different colorectal adenomas/cancer cohorts, and sebacic acid transferred well in all 3 datasets analyzed (Additional file 3: Figure S6). In some of these cases, we additionally observed a substantial overlap in significant contributors between the healthy and disease models (Additional file 3: Figure S7). In contrast, several metabolite models did not transfer well even though the metabolite was well-predicted in both the healthy dataset and linked disease dataset independently, potentially indicating a shift in microbiome metabolism in disease (Additional file 3: Figure S6; Additional file 3: Figure S7). The full catalog of significant contributors to both the healthy and the disease models can be found in Additional file 1: Table S7.

## Methods

### Data acquisition

In this study, we acquired data from several published studies of the human gut microbiome and metabolome. We focused on studies that included at least 40 individuals in each study group (or total, in non-case-control studies), for which both the microbiome and the metabolome were profiled from fecal samples. In case-control studies, cases and controls were treated as separate datasets to avoid the confounding impact of the disease state on the composition of both the microbiome and metabolome. Metabolomic datasets with only metabolite m/z info and without identification by name, KEGG IDs [62], or HMDB IDs [49] were discarded. For microbiome data, we considered both 16S rRNA gene sequencing and WGSS datasets. For 16S rRNA gene studies, we obtained raw fastq files and for WGSS datasets we obtained MetaPhlAn2 tables (see Additional file 1: Table S2 for additional information). For longitudinal datasets, where multiple samples were collected from each participant over time, we randomly chose up to 3 samples per subject, to avoid having a few subjects potentially dominating the dataset. Datasets were either downloaded from public repositories (e.g. NCBI Sequence Read Archive, Qiita [63]) or shared by the corresponding authors. As to June 2020, we identified over 70 published microbiome-metabolome studies of the human gut (reflected by fecal samples specifically), approximately 40 of which contained a sufficient number of samples as noted above. Of these, we were able to obtain the necessary data and metadata from only 10 datasets (either from online repositories or through the authors). Table 1 and Additional file 1: Table S1 describe the included datasets in detail and reference their corresponding publications. Since the difference between infants’ and adults’ microbiomes may introduce a particularly prominent source of heterogeneity, we also conducted a supplementary analysis, excluding the one infant dataset included in our study (the HE dataset). Overall, this exclusion did not markedly impact our findings and its results are reported in Additional file 2: Supplementary Note 4.

### Pre-processing of metabolomics datasets

We obtained processed metabolomics datasets from each study. As metabolomics technology, processing method, and output differed substantially between studies (see Additional file 1: Table S1 and S4), we pre-processed the metabolome datasets to allow comparison of metabolite-related findings across datasets. Importantly, we focused on unifying metabolite identifiers, and did not attempt to unify the measured values themselves. Specifically, we mapped metabolite identifications in each dataset to HMDB IDs. In many datasets, metabolites in the obtained data were identified by their names only, and we mapped metabolites to HMDB identifiers using MetaboAnalyst’s compound ID conversion utility [64]. Additional manual curation was performed in order to map metabolite names that were not identified by MetaboAnalyst (due to, for example, typos or special characters in the name, or in cases where MetaboAnalyst found more than one match). In datasets where metabolite masses were available, manual curation of such metabolites was also validated by mass.

To track cases where metabolite mappings to HMDB identifiers could not be done with high-confidence, we flagged metabolite annotations as “representative” in any of the following cases: Metabolites were marked as “representative” or “low-confidence annotation” in the original data; Annotation was ambiguous (e.g., due to mass spectrometry inability to differentiate two or more metabolites with the same mass and retention time); When the mapping was not automatic by MetaboAnalyst due to some uncertainty (e.g., when the metabolite name was listed with a different spelling); Or when there was some conflict between MetaboAnalyst mappings and original mappings. These “representative” annotations are marked in the supplementary tables and in Fig. 3A to indicate lower confidence.

Finally, metabolites present in 10 or fewer samples in each cohort were discarded, missing or zero values were replaced with 90% of the minimal metabolite concentration observed across all other samples, and metabolite values were log-transformed and scaled to zero-mean and unit-variance, as commonly accepted for such data [65]. Additional file 1: Table S4 details which data was available in each dataset and how the mapping was conducted.

### Pre-processing of 16S datasets

16S rRNA gene sequencing raw data was processed using QIIME2 version 2019-1 [48] as follows. When raw data was multiplexed, we demultiplexed the data using QIIME2’s demux plugin. We applied DADA2 [66] in order to denoise the data and extract amplicon sequence variants (ASVs). Whenever a sufficient high-quality overlap of forward and reverse reads was available, DADA2 was also used for merging paired end reads. We trimmed reads in each dataset to the first position with a median quality score under 30. In cases where this resulted in reads shorter than 100 base pairs, reads were trimmed according to the original publication.

To assign ASVs to taxonomy, we trained a Naive Bayes classifier per dataset using QIIME2’s feature-classifier plugin [67]. Classifiers were trained on reads extracted from the SILVA 99-OTU database [68], according to the specific 16S rRNA gene hypervariable region used in each dataset. We then collapsed the ASV table to genus-level counts, joining all reads without a genus-level annotation to an “Unclassified” category. This step was performed in order to make genera “entities” as comparable as possible between 16S-based and WGSS-based taxonomic profiles, given the substantial methodological gaps. In each dataset, samples with less than 1000 reads were removed. Read counts were normalized to sum to 1 in each sample, resulting in a table of relative abundances. Lastly, we removed rare genera, defined by either less than 25% non-zero values, or less than 10 non-zero values, or a mean relative abundance over samples of less than 0.1%. Further study-specific parameters and details about the 16S rRNA gene data processing of each dataset are detailed in Additional file 1: Table S2.

### Pre-processing of WGSS datasets

For WGSS datasets, we obtained taxonomic relative abundance tables precomputed by MetaPhlAn2 [69], with one exception where another detailed taxonomic profile was available (see Additional file 1: Table S2). In order to unify the WGSS and 16S rRNA-based taxonomic profiles, non-bacteria entities were discarded, all data was collapsed to genus-level relative abundances, and genera names were translated when necessary to the genera names used in the 16S rRNA gene processed data. Rare genera were removed from each dataset using the same criteria described above. All downstream analyses were performed on this unified genus-level relative abundance data. Further dataset-specific details about the processing of each WGSS data and unification of genus-level taxonomic annotations are detailed in Additional file 1: Tables S2 and S3.

### Implementation of a machine learning pipeline for predicting metabolite levels based on genus abundances

We implemented a machine learning pipeline to estimate how well genus-level profiles can predict metabolite levels in each dataset and for each metabolite. We specifically tried 4 pipeline settings: Random forest (RF) regression with default hyperparameters, RF regression with hyperparameters tuning, elastic net (ENet) with default hyperparameters (effectively making it a lasso regression), and ENet with hyperparameters tuning. We intentionally focused on relatively simple and commonly used machine learning models (as opposed to top-performing and much more complex models such as XGBoost [70]) to avoid the risk of increased overfitting. A comparison of pipeline results and further details are presented in Additional file 2: Supplementary Note 1. We eventually focused on results from the first pipeline, as it yielded the overall highest number of well-predicted metabolites.

RF regressors were trained for each HMDB-annotated metabolite in each dataset, as long as the metabolite appeared in 3 or more datasets overall. The performance of each such model was estimated using leave-one-out cross validation (LOOCV) to maximize the amount of data for training. In datasets where multiple samples existed per subject, we used a leave-one-subject-out approach and estimated performance using only a single sample from each out-of-fold subject. We calculated the RMSE, *R*^2^, and Spearman’s correlation coefficient, *ρ*, between actual metabolite levels and predicted ones for out-of-fold samples. Spearman’s correlation *P* value was also recorded, and FDR was applied to all metabolite-models in each dataset separately. Due to the randomness introduced by the RF models, we ran the entire pipeline 5 times per metabolite, verified that results are sufficiently stable (see Additional file 2: Supplementary Note 1), and reported performance metrics that were averaged over these 5 independent runs. We defined metabolites with a Spearman’s *ρ* > 0.3 and an FDR < 0.1 as “well-predicted” metabolites, similar to previous related works [33]. Lastly, we compared the number of well-predicted metabolites in each dataset to the number of well-predicted metabolites in a shuffled dataset, where metabolite values were shuffled across samples.

The above pipeline was implemented in R using the “tidymodels” package suite [71]. Specifically, we used “ranger” for RF [72] algorithm and “glmnet” for ENet [73].

### Defining robustly well-predicted metabolites

Meta-analysis is a well-established statistical framework for synthesizing results from multiple studies addressing the same topic [54]. Here, in order to synthesize results (correlation coefficients of predicted vs. actual metabolite levels) from multiple datasets, we adopted a classic meta-analysis statistical method, namely, random-effects models (REMs). REMs consider the effect sizes from each study, weighed proportionally to the study’s sample size, and assume that in addition to within-study variance, there is true heterogeneity between trials (i.e., between-study variance), likely resulting from differences in study settings and cohort characteristics. True effects are further assumed to follow a normal distribution, with an average effect *μ* and variation *τ*^2^. In our case, we chose REMs as we assumed that multiple factors may be affecting the predictive power of microbiota composition on metabolite levels, ranging from technical factors such as sample collection, storage, sequencing, or metabolomics instruments and processing, to cohort characteristics such as ages, diets, geographic locations, health status, and others.

Specifically, for each of the metabolites that appeared in 3 or more datasets, we used a REM to estimate the distribution of “true effects”, i.e., the predictability level of that metabolite based on genus-level profiles of the microbiota. We used the DerSimonian-Laird (DL) estimator for between-study variance, as implemented in the “meta” R package [74]. From each REM we then recorded the estimated average effect *μ*, its confidence interval, and its associated *P* value. The estimated average effect of a specific REM can be roughly interpreted as the average predictability of this metabolite over a new set of microbiome-metabolome studies. Smaller *P* values indicate higher confidence that the mean effect is indeed robust across a wide range of studies and cohorts. FDR correction was applied to all REM *P* values. Following our definition of “well-predicted” for a specific metabolite in a specific study above, we defined metabolites with a REM average effect > 0.3 and FDR < 0.1 as “robustly well-predicted” by the microbiome. Notably, different estimators for between-study variance (i.e., alternatives to the DL estimator) result in slightly different lists of robustly well-predicted metabolites. Additional file 1: Table S6 includes the full DL-based REM results, as well as information about which metabolites are robustly well-predicted when using alternative estimators.

Finally, to assess the obtained overall number of robustly well-predicted metabolites, we shuffled metabolite labels within each dataset 1000 times. For each shuffled dataset, we then ran the REM pipeline described above and re-computed the number of robustly well-predicted metabolites.

All REM computations were conducted using the “meta” [74] and “metafor” [75] R packages.

### Comparison of metabolite models in healthy datasets

For each robustly well-predicted metabolite that was also well-predicted independently in 3 or more datasets, totaling 41 metabolites, we used two analysis approaches to explore how similar are metabolite models trained on different datasets. For all of the below analyses, we only considered datasets in which the metabolite was well-predicted according to the definitions above.

In the first approach, we calculated permutation-based feature importance scores for each genus feature in each model using R’s ranger package [72]. We additionally applied the method introduced by Altman et al. (2010) to compute a *P* value for each feature in each model [76], and considered features with a *P* value below 0.1 as significant contributors. We then compared pairs of models by examining the number of shared significant contributors.

In the second analysis for assessing similarity between datasets, we tested how well a model trained on one dataset predicts metabolite levels in another dataset and vice versa, following previous microbiome-related meta-analysis studies [25, 77, 78]. We refer to this analysis as “cross-predictability analysis”. To overcome the differences between each pair of studies, we kept only genus features shared between both datasets, and additionally down-sampled the larger dataset to meet the sample size of the smaller dataset. After taking only the shared features, we re-normalized genus profiles to relative abundances. We then re-trained a RF regression model for the examined metabolite using the first dataset and evaluate its performance using LOOCV. If, due to down-sampling, the metabolite was no longer considered “well-predicted” by the new model, we discarded the specific comparison. Otherwise, we evaluated how well does the model perform on the second dataset and whether the performance meets the “well-predicted metabolite” definition used previously (i.e., the model transfers well). Due to the randomness presented by the down-sampling procedure and the RF itself, the final performance measures we report are averaged over 10 independent runs of the pipeline.

### Analysis of consistent genus contributors to metabolite models

We identified genera that consistently contributed to the models of a specific metabolite by calculating the number of models in which a genus was a significant contributor (as previously defined). If a genus significantly contributed to over half of the metabolite models it appeared in, we referred to the genus as a consistent contributor to that metabolite.

### Subgroup meta-analysis of healthy vs. disease datasets

Subgroup analysis is the process of comparing an effect (here, predictability of a metabolite) between two or more cohort “variants”. It is often used when some effect is suspected to differ between population subgroups (e.g., children vs. adults). Importantly, while large-scale studies often analyze these differences inherently, subgroup meta-analysis allows comparison of effect sizes between subgroups in smaller-scale studies and even when these subgroups appear in separate studies [54]. Here, we analyzed all metabolites with at least 3 healthy datasets and 3 disease datasets. We specifically used a REM for computing the average effect within subgroups and a fixed-effects model for estimating the between-subgroup differences. For each subgroup meta-analysis, we then recorded the mean predictability (and associated *P* value) within healthy datasets (as before), mean predictability (and associated *P* value) within disease datasets, and the *Q* statistic and *P* value for differences between subgroups. Sub-group meta-analysis was conducted using the “meta” [74] R package, based on code from the “dmetar” [79] package.

### Comparison of metabolite models in case-control datasets

As described before for comparison of models across healthy datasets, we used two analysis approaches to explore how similar are metabolite models trained on healthy vs. disease datasets from the same study. First, we compared significant contributors as previously described. Second, we performed a cross-predictability analysis to examine how well the healthy-model predicts metabolite levels in the pertaining disease dataset. To account for different study group sizes, the larger group was down-sampled to match the size of the smaller group. If, due to down-sampling, the metabolite was no longer well-predicted in the healthy group, we discarded that specific comparison. We limited our analysis to 5 case-control datasets that also had at least 1 well-predicted metabolite, and ran the entire analysis pipeline 10 times, averaging the results over these runs.

## Discussion

Understanding how the gut microbiome shapes the gut metabolome is without a doubt crucial for any investigation of microbiota-related mechanisms affecting the host’s health and of microbiome-based therapy. Yet, the interaction between gut microbes and metabolites remains largely uncharacterized for various technical and methodological reasons [3, 13]. Microbiome-metabolome studies of the human gut aim to characterize these interactions using a data-driven approach, but the generalizability of reported associations is unclear given the substantial differences between studies and cohorts.

Here we perform a first large-scale meta-analysis of paired fecal microbiome-metabolome datasets. We specifically evaluated the robustness of human gut microbiome-metabolite associations in 9 datasets of healthy individuals totaling 779 samples. We implemented a bioinformatic pipeline for processing these paired microbiome-metabolome datasets, used machine learning to predict metabolite levels in each dataset based on microbiome composition, and leveraged classic meta-analysis techniques to identify metabolites that are consistently well-predicted by the microbiome. Overall we found 97 such “robustly well-predicted” metabolites, spanning several known microbiome-related metabolic pathways such as bile acids [55], tryptophan metabolites [80], polyamines [81], and polyunsaturated fatty acids [82]. Several other metabolites, such as certain MCFAs and dicarboxylic acids, however, were barely reported in the context of the gut microbiota to date, and may suggest interesting and clinically relevant future research directions.

We further analyzed how similar are metabolite models across different datasets, both by comparing significant microbiota contributors in each model and by examining cross-predictability. We found that for some metabolites models were highly similar, yet for others, models exhibited low similarity across datasets and poor transferability. Lastly, we performed a similar analysis comparing the healthy datasets to 7 additional disease datasets and found that the overall predictability of most metabolites remained similar in disease.

Notably, the datasets included in this meta-analysis differ from one another in multiple aspects, both technical and biological. Technical factors include sample handling, sequencing/metabolomics methods, and bioinformatic methods for processing raw data, all of which were previously shown to contribute to artefactual differences in detected species or metabolites [2, 50, 51, 83, 84]. Metabolome profiling is particularly sensitive to technical variation in the profiling procedure, where the number of quantified metabolites can range from a few dozens to tens of thousands depending on whether a targeted or untargeted approach was taken and the exact instrumental setup used [2]. In our meta-analysis, the biases of each processing pipeline are potentially reflected in microbes and metabolites missing from the data, mislabeled, or in skewed abundance values. This sort of “noise”, however, is expected to mostly conceal associations, rather than to produce false ones. The second class of inter-study differences, i.e., those that stem from biological factors, include cohort characteristics such as age, geography, gender, medical background, and diet, all of which potentially introduce additional variation between datasets both in microbiome and metabolome compositions, and in microbiome-metabolome-host interactions [28, 51, 53, 85, 86]. Still, the goal of our study was to quantify variation in microbiome-metabolite associations that arise from all types of heterogeneity, and accordingly, to highlight those specific associations that seem to be consistent over all such heterogeneity-introducing factors. With the collection and publication of many additional microbiome-metabolome datasets, stratified analyses efforts could potentially reveal more robust associations while controlling for a specific factor of interest.

Beyond the findings reported in this study, our work calls attention to several important topics concerning microbiome-metabolome studies. First, the difficulties in processing and converting the different datasets into a unified format highlights a challenging first hurdle in any attempt to generalize findings across microbiome-metabolome studies. These difficulties arise both from the large variation in data formats, especially for metabolomics data, and from the substantial differences in detected microbiome/metabolome features in each study as discussed above. Indeed, previous meta-analyses studies have discussed these challenges in the context of microbiome or metabolome independently [47, 87–89] and non-surprisingly these challenges further exacerbate here in this multi-omic meta-analysis. Improved standardization in collecting, processing, cataloging, and storing both microbiome and metabolome data (and maybe even specific standards for microbiome-metabolome datasets) is therefore key for future progress in this field.

We also note that in the context of case-control studies, where researchers attempt to detect microbiome-metabolite links characteristic of a specific disease, there may be a few different types of potential disease-relevant patterns. First, a metabolite may be strongly linked to the overall microbial composition (e.g., well-predicted) in both the case and the control study groups, but enriched or depleted in one of the groups. Such a pattern (especially if this microbiome-metabolite link is found also in many other studies) may suggest that this specific association reflects a part of the basic “house-keeping” metabolic machinery of the microbiome and that the shift in the metabolite level in disease may be attributed to a dysbiotic microbiome. Second, a metabolite may be well-predicted by the microbiome in both groups but with different taxa contributors in each group, suggesting again an altered composition or an altered metabolic activity of community members in disease. Lastly, a metabolite may be well-predicted in one group (e.g., control), but not the other. In this case, that metabolite’s level in individuals with the disease is potentially controlled more by other disease-related factors. Overall, this suggests that the robustness of microbiome-metabolite links, both across studies and across study groups, should be taken into consideration when interpreting disease-associated shifts and calls for a better theory and methodology for distinguishing between clinically relevant and non-relevant cross-omic interactions.

Most microbiome-metabolome studies conduct some sort of an association-based analysis, using either correlations or simple linear regressions to identify specific microbes whose abundances across samples strongly correlate with the concentration of a specific metabolite. A smaller subset of such studies further apply machine learning techniques for this purpose, and new machine learning-based tools have been recently introduced [20, 33, 34, 41, 90, 91]. In this work, we intentionally opted for this less common, machine learning-based approach, treating the ability to predict a metabolite level based on the composition of the microbiome as a quantifiable proxy for metabolite-microbiota associations. The motivation for this choice was two-fold: first, machine learning enables to detect non-linear and complex associations that may involve multiple different factors and that may be missed in a simpler, correlation-based approach; second, by considering the entire microbial community, rather than a specific taxon abundance, we were able to identify metabolites consistently associated with the microbiota (or a sub-population of it) as a whole while overcoming the substantial differences between studies in terms of taxonomic resolution and accuracy that potentially prevent meaningful taxon-specific comparisons. Still, as both machine learning and univariate methods ultimately capture statistical dependencies in the data, we believe that many of the insights reported in our study are relevant for other statistical methods such as correlation.

Importantly, though, high predictability of a metabolite does not necessarily indicate a direct mechanistic interaction between the microbes that contribute toward this prediction and the metabolite, just as a microbe-metabolite correlation does not necessarily indicate a direct relation of consumption or production [36]. Nonetheless, these statistical associations reveal potentially intriguing structures and dependencies in the data and so their robustness and generalizability are key. We hypothesize that the metabolites found here as robustly well-predicted are strongly tied to the gut microbiota composition even in the presence of other influencing factors, and hence that changes in the microbial composition will most likely cause changes in metabolite levels, making such metabolites a prime target for microbiome-based interventions.

Our analysis clearly faced several important caveats. One major challenge of this study and of the need to unify data from multiple different studies is that we had to limit our analysis to metabolites with a shared annotation (here, HMDB identifiers) and that are present in multiple studies (using potentially different metabolomic approaches). With these limitations, we effectively focused on 273 metabolites out of approximately 97,000 (non-unique) raw metabolites identified in the 9 healthy datasets. These 273 metabolites constitute only a tiny fraction of gut metabolites, as most gut metabolites, and specifically those of microbial origin, are largely unidentified and poorly represented in metabolic databases [3, 17, 92]. Moreover, though for the main part of the analysis, we did not directly compare genus-level statistics between cohorts, a similar limitation is applicable for the microbiome data. Collapsing the taxonomic data into genus level profiles somewhat mitigated this problem, but also constrained our ability to capture associations at the species and strain level [93–95]. Our observation that some genera are consistently associated with metabolites while others demonstrate inconsistent patterns, for example, may be affected by the extent of metabolic diversification within each genera, which is known to vary dramatically [96]. This meta-analysis was also clearly limited by the number of cohorts included. Future work including significantly more cohorts could not only substantially expand the pool of analyzed metabolites but could also better account for technical and biological differences between studies, as noted above. Furthermore, expanding the analysis to multiple “disease” cohorts, perhaps even of the same disease, could uncover additional insights related to consistent shifts in metabolic activity of dysbiotic microbiota. This calls for both additional high-quality microbiome-metabolome studies and improved availability and standardization of such datasets.

## Conclusions

The network of host-microbiota-metabolome interactions in the human gut is extremely complex and requires multiple research efforts in multiple complementary directions in order to be fully deciphered and characterized. Here, we identified metabolites consistently associated with the microbiota across diverse studies and cohorts, thus distinguishing between study-specific and robust or universal links. Our findings provide a better understanding of microbiome-metabolome interactions and allow researchers to put newly identified microbially associated metabolites within the context of other studies.

## Supplementary Information

**Additional file 1: Supplementary Table S1.** Description of studies included in the analysis (expansion of Table 1). **Table S2.** 16S/WGSS data processing notes per dataset. **Table S3.** Unified genera processing notes. **Table S4.** Metabolomics processing notes per dataset. **Table S5.** Metabolite predictability per dataset. **Table S6.** Random-effects models results. **Table S7.** Genus feature contributions by permutation analysis. **Table S8.** Consistent genera contributors. **Table S9.** Subgroup meta-analysis results.

**Additional file 2: Supplementary Note 1.** Alternative machine learning pipelines and effect on results. **Supplementary Note 2.** Comparisons to previous studies and validation. **Supplementary Note 3.** Metabolic pathways-oriented analysis of robustly well-predicted metabolites. **Supplementary Note 4.** Effect of excluding the infants dataset on robustness results.

**Additional file 3: Figure S1.** Statistics about genera and metabolite features shared among datasets included in this meta-analysis. **Figure S2.** Comparisons to previous studies. **Figure S3.** Characteristics of robustly well-predicted metabolites. **Figure S4.** Comparisons of metabolite models between datasets. **Figure S5.** Examples of disease-independent robustly well-predicted metabolites. **Figure S6.** Transferability of metabolite models from controls to cases within the same study. **Figure S7.** Examples of significant contributors’ comparison between healthy and disease metabolite models. **Figure S8.** Comparison of different machine learning pipelines.

## Data Availability

Raw or processed sequencing and metabolomics data for each study included in the analysis can be accessed as described in Additional file 1: Table S1. Detailed predictability results, meta-analysis random-effects model results, and additional analysis results reported throughout the paper are available in the supplementary files. R scripts used for analysis are available on https://github.com/borenstein-lab.
