# Supplementary material for: A meta-analysis study of the robustness and universality of gut microbiome-metabolome associations
Source: Microbiome. 2021 Oct 12;9:203. doi: 10.1186/s40168-021-01149-z (PMC8507343; doi:10.1186/s40168-021-01149-z)
Supplement: Supplementary file 3 — Additional file 2: Supplementary Note 1. Alternative machine learning pipelines and effect on results. Supplementary Note 2. Comparisons to previous studies and validation. Supplementary Note 3. Metabolic pathways-oriented analysis of robustly well-predicted metabolites. Supplementary Note 4. Effect of excluding the infants dataset on robustness results. [file 40168_2021_1149_MOESM3_ESM.pdf]

Supplementary Notes

Supplementary note 1: Alternative machine learning pipelines and effect on results

In order to find a machine learning pipeline that best fits our multi-study data, we examined several different pipelines, and eventually chose the one that produced the highest number of well-predicted metabolites overall among the healthy datasets.

Specifically, we tried both random forest (RF) and elastic net models (ENet), from the "ranger" [1] and "glmnet" [2] packages, respectively. For each algorithm, we additionally tried both a pipeline that included model tuning via grid search and a pipeline that did not include tuning and used default hyperparameters instead. For the tuned pipeline, we used nested cross-validation (CV), meaning that an outer 10-fold CV loop was used for estimating the overall model performance, and an inner 10-fold CV loop was used to find hyperparameters that optimize the root mean square error (RMSE). The hyperparameters we tuned for RF were *mtry* (number of features per tree), *trees* (number of trees), and *min_n* (minimal number of samples in a tree node). For ENet, we tuned *mixture* (proportion of L1 regularization in the model) and *penalty* (total amount of regularization in the model) hyperparameters. Each pipeline produced qualitatively similar results in terms of which metabolites were most predictable but some differed in the final set of well-predicted metabolites given our strict cutoff (Additional file 3: Figure S8). Overall, the RF models performed better on our data than the ENet ones, with highly similar results between the pipeline version with tuning and that without tuning (Additional file 3: Figure S8). The RF-without-tuning yielded 418 well-predicted metabolites out of 1255 models (accumulating over all datasets), and the RF-with-tuning, ENet-with-tuning, and ENet-without-tuning yielded 407, 323, and 241 well-predicted metabolites, respectively.

We ran each regression task (a specific metabolite in a specific dataset) 5 times to also examine the stability of each pipeline, given the randomness introduced by the models. All pipelines were similarly stable, as quantified by the percent of metabolites that were either always well-predicted or never well-predicted across the 5 runs. Specifically, all pipelines resulted in 83%-86% metabolites being always or never well-predicted, and 92%-95% if also allowing 1 of the 5 runs to disagree with the others.

The pipeline that resulted in most well-predicted metabolites overall was the one without hyperparameter tuning using RF with default hyperparameters and was thus selected for further analysis.

Supplementary note 2: Comparisons to previous studies and validation

As a validation of our machine learning pipeline, we compared the predictability of metabolites obtained using our pipeline in one specific dataset to results from a recent study that applied a somewhat different machine learning pipeline to the same dataset. In this study (Mallick *et al.* [3]), a machine learning method, termed *MelonnPan*, was used for predicting metabolite levels based on functional profiles of the microbiome. Mallick *et al.* used a dataset of IBD patients and controls to train and tune the models, and another independent cohort to evaluate performance and determine which metabolites can be well-predicted (using a similar threshold to the one used in our study). The combined dataset from that study is also included in our meta-analysis and is labeled as 'FRANZOSA_IBD'. Importantly, while both MelonnPan and our study use a machine learning-based framework, a few important differences should be acknowledged. First, Mallick *et al.* trained the model on a mix of IBD and control subjects from the training set, while in our study we considered both the training and validation cohorts but *only* the healthy subjects. Second, Mallick *et al*. used gene-family relative abundances as features, whereas our study used genera relative abundances. Yet, even when considering these methodological differences, since both studies ultimately aimed to predict metabolite levels based on microbiome composition, we expected a significant overlap. To compare our results with those reported in Mallick *et al.*, we obtained the list of 107 well-predicted metabolites from that study's supplementary data, and mapped metabolite names to HMDB IDs using MetaboAnalyst [4]. Out of these 107 metabolites, 98 were mapped to HMDB IDs, of which 81 were also included in our analysis. We found that 60 metabolites (74%) of these 81 were also well-predicted by our pipeline in this dataset (Additional file 3: Figure S3A). Interestingly, 20 of the 21 metabolites well-predicted in Mallick *et al.* but not well-predicted by our pipeline, were also significantly associated with IBD (each metabolite tested independently using a Mann-Whitney test, with FDR-corrected P value < 0.05), suggesting that the disease status may have amplified the predictability of these metabolites when training the machine learning models on mixed case-control datasets.

Finally, to also provide additional support to our final set of *robustly* well-predicted metabolites, we compared this set to findings from an independent analysis [5] of paired stool microbiome-metabolome profiles from the TwinsUK cohort [6] – the largest cohort with such data published to date (not included in our meta-analysis due to limited data availability). As part of this analysis, the authors have estimated the proportion of variance in each detected metabolite explained by the composition of the microbiota, by regressing fecal metabolite concentrations against the microbial UniFrac beta-diversity. Since we believe that our set of *robustly* well-predicted metabolites captures consistent, robust, and reproducible associations between the microbiome and specific metabolites, we expected this set to show some agreement with microbiome-metabolite associations detected by a completely different

statistical framework and in a new, independent dataset. Indeed, obtaining the calculated estimations of explained variance from this study, we found that robustly well-predicted metabolites were associated with significantly higher proportions of variance explained compared to metabolites not in this set (Mann-Whitney P = 0.0008, Additional file 3: Figure S3B).

Supplementary note 3: Metabolic pathways-oriented analysis of robustly well-predicted metabolites

Among robustly well-predicted metabolites, we found multiple metabolites that take part in clinically important pathways known to involve the gut microbiome, such as bile acid transformations, trimethylamine N-oxide (TMAO) metabolism, tryptophan metabolism, and polyamine biosynthesis. Here, we elaborate on several of these metabolic pathways, highlighting which related metabolites were robustly associated with the microbiome and how these findings coincide with existing literature.

Bile acids have an essential role in fat digestion but also act systematically as hormones, regulating both glucose and fatty acids levels and immune homeostasis [7]. Our analysis echoes the microbiota's known essential role in the transformation of primary bile acids to secondary bile acids [7]. Specifically, we found that the levels of the primary bile acids cholic acid (HMDB0000619), chenodeoxycholic acid (HMDB0000518), glycochenodeoxycholate (HMDB0000637) taurocholic acid (HMDB0000036), taurodeoxycholic acid (HMDB0000896), lithocholyltaurine (HMDB0000722), and taurochenodeoxycholate (HMDB0000951), as well as secondary bile acids such as lithocholic acid (HMDB0000761), were all robustlywell-predicted by the microbiota's composition. We additionally found that the Blautia genus consistently contributes to cholic acid models (Additional file 1: Table S8). Indeed, previously reported genomic analysis revealed that strains of this genus contain bile acid hydrolysis enzymes [7]. This finding is also supported by rodent models: Mice that were fed cholic acid showed a drastic increase in Blautia abundance [8]. Additional major bile components were robustly well-predicted, including cholesterol (HMDB0000067), taurine (HMDB0000251, a deconjugation product of the primary bile acid taurochenodesoxycholate), and urobilin (the oxidized form of urobilinogen, a product of microbial metabolism of bile pigment) (see Figure 3C). Contributors analysis indicated that Bacteroides played a consistent role in cholesterol models, consistent with previous reports [9, 10]. Our analyses also indicated that the Bilophila genus is consistently associated with Taurine, again in agreement with experimental results [11, 12].

TMAO metabolism by both the host and gut bacteria has been widely studied for its involvement in atherosclerosis development. Specifically, dietary choline and L-carnitine are metabolized by intestinal bacteria to produce TMA, which, in turn, is further oxidized into TMAO in the liver and absorbed into the bloodstream [13, 14]. Two metabolites involved in this process were found to be robustly well-

predicted, namely N6, N6, N6-Trimethyl-L-lysine (HMDB0001325), and gamma-butyrobetaine (HMDB0001161, an intermediate in gut microbe-dependent formation of TMA from L-carnitine) [14, 15]. TMAO itself was included in our analysis but was not robustly well-predicted, perhaps unsurprisingly as the majority of TMAO is extracted in urine and only a small fraction (~4%) is extracted in feces [16]. This most likely indicates that TMAO extraction in the stool is affected more by inter-personal physiological variation rather than differences in microbial activity in the gut.

Perhaps one of the most well-studied classes of microbially-governed metabolites is short chain fatty acids (SCFA). SCFAs have been shown to influence a wide spectrum of physiological processes, ranging from gut-brain axis crosstalk to immunomodulation [17]. However, quantification of SCFAs in common untargeted MS methods is challenging due to their high volatility [18]. As a result, most of the SCFAs are missing from our analysis, and only butyric acid was found to be robustly well-predicted.

Our analysis also supports the established microbial involvement in tryptophan metabolism [19]. Tryptophan itself as well as its derivatives, tryptamine and indolepropionate were all robustly well-predicted (see Additional file 3: Figure S3B). These two derivatives are known agonists of the aryl hydrocarbon receptor, an important transcription factor that mediates xenobiotic degradation and immune response [20, 21]. Our analysis found that members of the Odoribacter genus consistently contributed to tryptophan models, in line with evidence of *Odoribacter Splanchnicus*'s (isolated from human stool) ability to metabolize tryptophan [22].

Polyamines are ubiquitous to all living cells and possess a wide set of biological functions including gene regulation, resistance to oxidative stress, and cell proliferation and differentiation [23]. In certain cancers, polyamine metabolism is dysregulated, and several recent and ongoing clinical trials are testing agents targeting polyamines for both therapy and prevention of cancer [24]. The colonic bacterial population is known to directly contribute to shifts in polyamine metabolism (and therefore to carcinogenesis), and indeed levels of several polyamines and related metabolites in the stool were consistently associated with the microbiome in our analysis, including N1,N12-diacetylspermine (HMDB0002172), putrescine (HMDB0001414), N-acetylputrescine (HMDB0002064), S-adenosylmethionine (HMDB0001185), cadaverine (HMDB0002322) N1-acetylspermidine (HMDB0001276), and N1-acetylspermine (HMDB0001186) [23, 25] (see Additional file 3: Figure S3C). The Alistipes genus was a consistent contributor to all robustly well-predicted polyamines. Indeed, this bacteria was found to possess enzymes in the polyamines metabolic pathway [26].

Two robustly well-predicted metabolites, namely gamma-aminobutyric acid (GABA, HMDB0000112) and N-acetyl-L-aspartic acid (HMDB0000812), also illustrated the suggested role of the microbiome in the gut-brain axis. GABA is an important neurotransmitter that was found to be metabolized by

Bacteroides strains [27]. N-acetyl-L-aspartic acid is the most abundant amino acid in brain tissue and is a key osmolyte and precursor for the neurotransmitter N-acetylaspartylglutamate [28].

Our results additionally support more recent findings such as multiple commensal gut bacteria's role in L-proline biosynthesis, discovered using protein similarity networks [29], specifically by acting on 4-hydroxyproline (HMDB000025) which was robustly well-predicted in our analysis. 4-hydroxyproline can also be obtained through diet and carries health benefits [30].

Lastly, we note the class of polyunsaturated fatty acids (PUFA). PUFAs, and specifically omega-3 and omega-6 acids, all have a key role in regulating the homeostasis of the immune system, lipid metabolism, and inflammatory reaction and have an important role in cancer development, food allergies, and cardiovascular diseases [31]. Our analysis highlights the microbial role in the metabolism of PUFAs, including many omega-6 derivatives such as dihomo-gamma-linolenic acid (HMDB0002925), arachidonic acid (HMDB0001043), adrenic acid (also named docosatetraenoic acid, HMDB0002226), docosapentaenoic acid (22n-6) (HMDB0001976), 9,10-DHOME (HMDB0004704) and 12,13-DHOME (HMDB0004705). The analysis further indicates the association of gut microbes and the omega-3 acids eicosapentaenoic acid (HMDB0001999) and Docosahexaenoic acid (HMDB0002183). These PUFA-microbiota associations are supported by previous experimental findings, as metabolism of PUFAs was detected in cultured human intestinal bacteria [32]. In addition, experiments in specific pathogen-free mice compared to germ-free mice have established the role of mice gastrointestinal bacteria in modifying the fatty acid profiles of their hosts, in particular by increasing the levels of intermediates of polyunsaturated fatty acid-saturation metabolism [33]. Bacteroides genus was a consistent contributor to both docosapentaenoic acid (22n-6) and dihomo-gamma-linolenic acid models. This finding is supported by experiments in piglets, where piglets fed with omega 3 rich oils exhibited growth in cecum Bacteroides population compared to piglets fed with omega-6 rich oils [34].

Supplementary note 4: Effect of excluding the infants dataset on robustness results

While multiple sources of heterogeneity exist between the datasets included in this meta-analysis study (as discussed in the main text), the difference between infants' and adults' microbiomes may constitute one of the most prominent heterogeneity source. Specifically, the infant gut differs substantially from that of adults in both digestion, absorption, and motility [35, 36]. Moreover, microbial composition and metabolic activity in the gut changes dramatically over the first years of life [35, 37]. We therefore conducted an additional analysis in which we excluded the infants' dataset included in our study (HE_INFANTS or HE in short), and report results below.

Out of 940 unique, non-rare, HMDB compound IDs found across the 8 healthy datasets (compared to 951 when including HE), 264 (28%) were shared among 3 or more datasets. Training a predictor for each of the 264 metabolites and in each dataset it appeared in resulted in a total of 1161 metabolite predictor models. Of these, 401 models were able to successfully predict the metabolite level (with $\rho > 0.3$ and FDR < 0.1), and accordingly defined as well-predicted.

Using random-effects models and following the strategy and thresholds applied when analyzing the complete set of datasets, we found 97 robustly well-predicted metabolites, mostly overlapping with the 97 found when including HE_INFANTS dataset. Specifically, 93 of the robustly well-predicted metabolites remained so when excluding HE. Four metabolites that were found to be robustly well-predicted when excluding the HE dataset and were not robustly well-predicted when the HE dataset was included were L-alpha-Aminobutyric acid (HMDB0000452), L-Methionine (HMDB0000696), D-Xylose (HMDB0000098), and 3-Hydroxybutyric acid (HMDB0000011). For all of these metabolites, the performance of the microbiome-based models in the HE dataset was poor (Spearman correlation < 0.16 and FDR-corrected p value > 0.4), and thus, when excluding HE from the corresponding random-effects models, the overall mean predictability was higher and exceeded the defined cutoff for robustness. The four metabolites that were no longer robustly well-predicted after excluding HE were 2-Hydroxy-3-methylbutyric acid (HMDB0000407), gamma-Aminobutyric acid (HMDB0000112), L-Arabinose (HMDB0000646), and Creatine (HMDB0000064). The first 3 simply no longer appeared in 3 datasets and were therefor not included in the random-effects models analysis. Creatine did appear in enough datasets but without HE the overall predictability estimate dropped below our threshold. Though some of these metabolites may indeed interact differently with the microbiome in the infant gut compared to the adult gut, more infant datasets are required in order to more rigorously determine such differences.

References

1. Wright MN, Ziegler A. ranger: A Fast Implementation of Random Forests for High Dimensional Data in C++ and R. J Stat Softw. 2015;77. doi:10.18637/jss.v077.i01.

2. Friedman J, Hastie T, Tibshirani R. Regularization paths for generalized linear models via coordinate descent. J Stat Softw. 2010;33:1–22. doi:10.18637/jss.v033.i01.

3. Mallick H, Franzosa EA, Mclver LJ, Banerjee S, Sirota-Madi A, Kostic AD, et al. Predictive metabolomic profiling of microbial communities using amplicon or metagenomic sequences. Nat Commun. 2019;10:3136. doi:10.1038/s41467-019-10927-1.

4. Chong J, Soufan O, Li C, Caraus I, Li S, Bourque G, et al. MetaboAnalyst 4.0: towards more transparent and integrative metabolomics analysis | Nucleic Acids Research | Oxford Academic. Nucleic Acids Res. 2018;46:W486–94. https://academic.oup.com/nar/article/46/W1/W486/4995686. Accessed 4 Aug 2020.

5. Zierer J, Jackson MA, Kastenmüller G, Mangino M, Long T, Telenti A, et al. The fecal metabolome as a functional readout of the gut microbiome. Nat Genet. 2018;50:790–5. doi:10.1038/s41588-018-0135-7.

6. Spector TD, Williams FMK. The UK Adult Twin Registry (TwinsUK). Twin Res Hum Genet. 2006;9:899–906.

7. Molinero N, Ruiz L, Sánchez B, Margolles A, Delgado S. Intestinal Bacteria Interplay With Bile and Cholesterol Metabolism: Implications on Host Physiology. Front Physiol. 2019;10 MAR:185. doi:10.3389/fphys.2019.00185.

8. Islam KBMS, Fukiya S, Hagio M, Fujii N, Ishizuka S, Ooka T, et al. Bile acid is a host factor that regulates the composition of the cecal microbiota in rats. Gastroenterology. 2011;141:1773–81.

9. Gérard P, Lepercq P, Leclerc M, Gavini F, Raibaud P, Juste C. Bacteroides sp. strain D8, the first cholesterol-reducing bacterium isolated from human feces. Appl Environ Microbiol. 2007;73:5742–9. doi:10.1128/AEM.02806-06.

10. Kriaa A, Bourgin M, Potiron A, Mkaouar H, Jablaoui A, Gérard P, et al. Microbial impact on cholesterol and bile acid metabolism: current status and future prospects. Journal of Lipid Research. 2019;60:323–32. doi:10.1194/jlr.R088989.

11. Laue H, Friedrich M, Ruff J, Cook AM. Dissimilatory sulfite reductase (Desulfoviridin) of the taurine-degrading, non-sulfate-reducing bacterium Bilophila wadsworthia RZATAU contains a fused DsrB-DsrD subunit. J Bacteriol. 2001;183:1727–33. doi:10.1128/JB.183.5.1727-1733.2001.

12. Laue H, Denger K, Cook AM. Taurine reduction in anaerobic respiration of Bilophila wadsworthia RZATAU. Appl Environ Microbiol. 1997;63.

13. Wang Z, Klipfell E, Bennett BJ, Koeth R, Levison BS, Dugar B, et al. Gut flora metabolism of phosphatidylcholine promotes cardiovascular disease. Nature. 2011;472:57–65. doi:10.1038/nature09922.

14. Koeth RA, Wang Z, Levison BS, Buffa JA, Org E, Sheehy BT, et al. Intestinal microbiota metabolism of l-carnitine, a nutrient in red meat, promotes atherosclerosis. Nat Med. 2013;19:576–85.

15. Rebouche CJ, Seim H. Carnitine metabolism and its regulation in microorganisms and mammals. Annu Rev Nutr. 1998;18:39–61.

16. Zeisel SH, Warrier M. Trimethylamine N-Oxide, the Microbiome, and Heart and Kidney Disease. Annu Rev Nutr. 2017;37:157–81.

17. Dalile B, Van Oudenhove L, Vervliet B, Verbeke K. The role of short-chain fatty acids in microbiota–gut–brain communication. Nat Rev Gastroenterol Hepatol. 2019;16:461–78. doi:10.1038/s41575-019-0157-3.

18. Li M, Zhu R, Song X, Wang Z, Weng H, Liang J. A sensitive method for the quantification of short-chain fatty acids by benzyl chloroformate derivatization combined with GC-MS. Analyst. 2020;145:2692–700.

19. Ma N, Ma X. Dietary Amino Acids and the Gut-Microbiome-Immune Axis: Physiological Metabolism and Therapeutic Prospects. Compr Rev Food Sci Food Saf. 2019;18:221–42.

20. Cheng Y, Jin UH, Allred CD, Jayaraman A, Chapkin RS, Safe S. Aryl hydrocarbon receptor activity of tryptophan metabolites in young adult mouse colonocytes. Drug Metab Dispos. 2015;43:1536–43.

21. Quintana FJ, Sherr DH. Aryl hydrocarbon receptor control of adaptive immunity. Pharmacol Rev. 2013;65:1148–61.

22. Göker M, Gronow S, Zeytun A, Nolan M, Lucas S, Lapidus A, et al. Complete genome sequence of odoribacter splanchnicus type strain (1651/6 T). Stand Genomic Sci. 2011;4:200–9. doi:10.4056/sigs.1714269.

23. Tofalo R, Cocchi S, Suzzi G. Polyamines and Gut Microbiota. Front Nutr. 2019;6:16. doi:10.3389/fnut.2019.00016.

24. Casero RA, Murray Stewart T, Pegg AE. Polyamine metabolism and cancer: treatments, challenges and opportunities. Nat Rev Cancer. 2018;18:681–95. doi:10.1038/s41568-018-0050-3.

25. Matsumoto M, Benno Y. The Relationship between Microbiota and Polyamine Concentration in the Human Intestine: A Pilot Study. Microbiol Immunol. 2007;51:25–35. doi:10.1111/j.1348-0421.2007.tb03887.x.

26. Lei M, Menon R, Manteiga S, Alden N, Hunt C, Alaniz RC, et al. Environmental Chemical Diethylhexyl Phthalate Alters Intestinal Microbiota Community Structure and Metabolite Profile in Mice. mSystems. 2019;4. doi:10.1128/msystems.00724-19.

27. Strandwitz P, Kim KH, Terekhova D, Liu JK, Sharma A, Levering J, et al. GABA-modulating bacteria of the human gut microbiota. Nat Microbiol. 2019;4:396–403. doi:10.1038/s41564-018-0307-3.

28. Baslow MH. Functions of N-acetyl-L-aspartate and N-acetyl-L-aspartylglutamate in the vertebrate brain: Role in glial cell-specific signaling. J Neurochem. 2000;75:453–9.

29. Levin BJ, Huang YY, Peck SC, Wei Y, Martínez-Del Campo A, Marks JA, et al. A prominent glycyl radical enzyme in human gut microbiomes metabolizes trans-4-hydroxy-L-proline. Science. 2017;355.

30. Wu G. Important roles of dietary taurine, creatine, carnosine, anserine and 4-hydroxyproline in human nutrition and health. Amino Acids. 2020;52:329–60. doi:10.1007/s00726-020-02823-6.

31. Saini RK, Keum YS. Omega-3 and omega-6 polyunsaturated fatty acids: Dietary sources, metabolism, and significance — A review. Life Sciences. 2018;203:255–67.

32. Devillard E, McIntosh FM, Duncan SH, Wallace RJ. Metabolism of linoleic acid by human gut bacteria: Different routes for biosynthesis of conjugated linoleic acid. J Bacteriol. 2007;189:2566–70. doi:10.1128/JB.01359-06.

33. Kishino S, Takeuchi M, Park SB, Hirata A, Kitamura N, Kunisawa J, et al. Polyunsaturated fatty acid saturation by gut lactic acid bacteria affecting host lipid composition. Proc Natl Acad Sci U S A. 2013;110:17808–13.

34. Andersen AD, Mølbak L, Thymann T, Michaelsen KF, Lauritzen L. Dietary long-chain n-3 PUFA, gut microbiota and fat mass in early postnatal piglet development-exploring a potential interplay. Prostaglandins Leukot Essent Fat Acids. 2011;85:345–51. doi:10.1016/j.plefa.2011.08.004.

35. Mackie RI, Sghir A, Gaskins HR. Developmental microbial ecology of the neonatal gastrointestinal tract. In: American Journal of Clinical Nutrition. American Society for Nutrition; 1999. p. 1035s-1045s. doi:10.1093/ajcn/69.5.1035s.

36. Commare CE, Tappenden KA. Development of the infant intestine: Implications for nutrition support. Nutrition in Clinical Practice. 2007;22:159–73. doi:10.1177/0115426507022002159.

37. Bittinger K, Zhao C, Li Y, Ford E, Friedman ES, Ni J, et al. Bacterial colonization reprograms the neonatal gut metabolome. Nat Microbiol. 2020;5:838–47. doi:10.1038/s41564-020-0694-0.
