# Supplementary material for: A meta-analysis study of the robustness and universality of gut microbiome-metabolome associations
Source: Microbiome. 2021 Oct 12;9:203. doi: 10.1186/s40168-021-01149-z (PMC8507343; doi:10.1186/s40168-021-01149-z)
Supplement: Supplementary file 4 — Additional file 3: Figure S1. Statistics about genera and metabolite features shared among datasets included in this meta-analysis. Figure S2. Comparisons to previous studies. Figure S3. Characteristics of robustly well-predicted metabolites. Figure S4. Comparisons of metabolite models between datasets. Figure S5. Examples of disease-independent robustly well-predicted metabolites. Figure S6. Transferability of metabolite models from controls to cases within the same study. Figure S7. Examples of significant contributors’ comparison between healthy and disease metabolite models. Figure S8. Comparison of different machine learning pipelines. [file 40168_2021_1149_MOESM4_ESM.pdf]

Figure S1

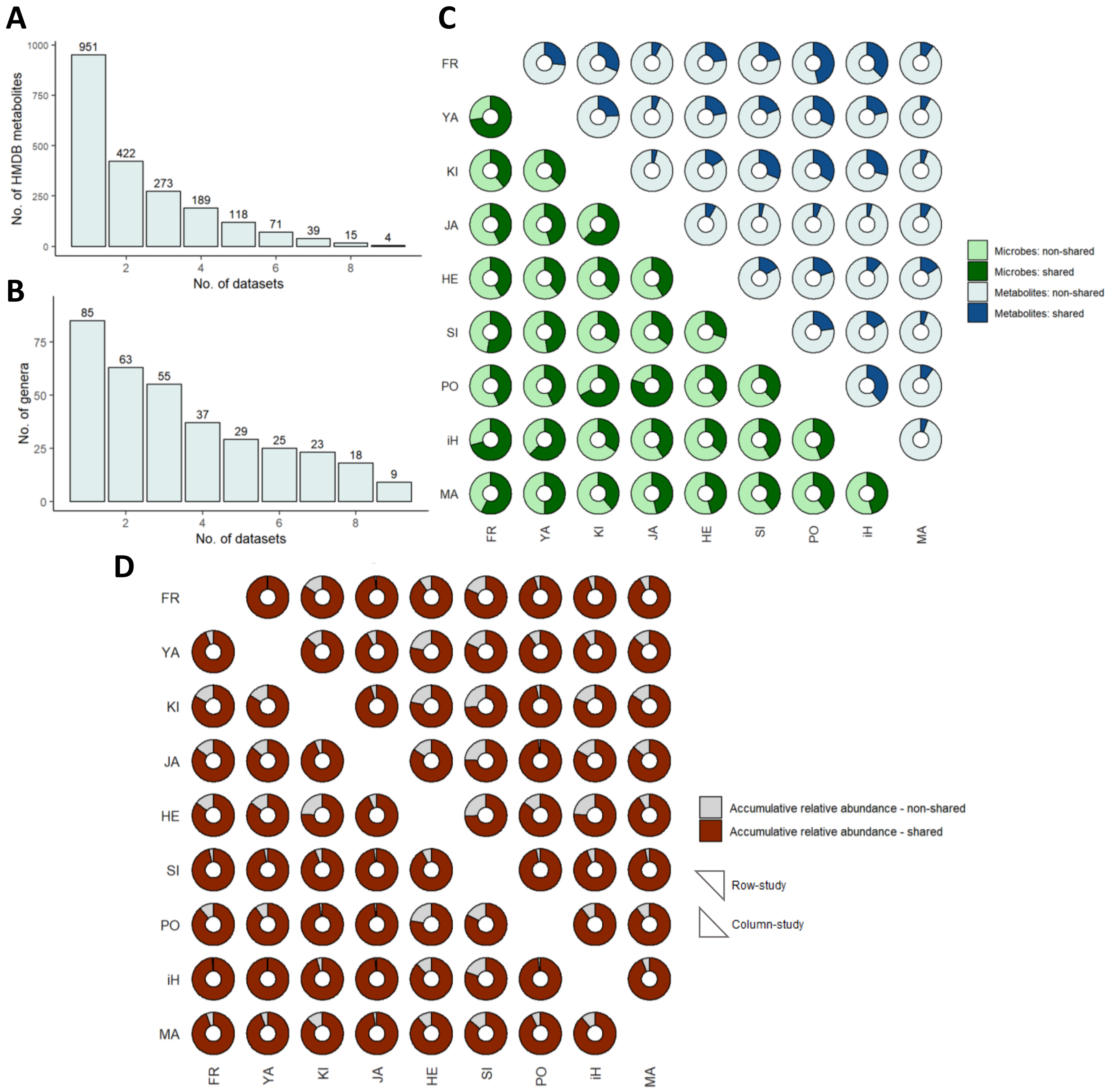
A
No. of HMDB metabolites
1000
750
500
250
0
951
422
273
189
118
71
39
15
4
2
4
6
8
No. of datasets
B
No. of genera
75
50
25
0
85
63
55
37
29
25
23
18
9
2
4
6
8
No. of datasets
C
FR
YA
KI
JA
HE
SI
PO
iH
MA
Microbes: non-shared
Microbes: shared
Metabolites: non-shared
Metabolites: shared
D
FR
YA
KI
JA
HE
SI
PO
iH
MA
Accumulative relative abundance - non-shared
Accumulative relative abundance - shared
Row-study
Column-study

Figure S1: Statistics about genera and metabolite features shared among datasets. (A) Bar-plot illustrating the cumulative number of metabolites shared by any number of datasets, i.e. each bar represents the number of metabolites shared by at least the specified amount of datasets. Only non-rare metabolites that were successfully mapped to HMDB identifiers are considered (see Methods). **(B)** Bar-plot illustrating the cumulative number of genera shared by any number of studies. Only non-rare genera were considered (see Methods). **(C)** Pairwise comparisons in feature overlap between datasets. Each donut in the matrix represents a pair of studies ("row-study" and "column-study"). Donuts on the upper right triangle represent unique metabolites in both studies, where the dark blue portion represents unique metabolites shared between both studies and light blue represents metabolites available in one dataset but not the other. Similarly, the lower left triangle represents genera overlap between studies, with dark green indicating shared genera and light green indicating non-shared genera. **(D)** Pairwise comparison of genera overlap, considering genera relative abundances in each dataset. As in panel C, each donut represents a pair of studies. In the upper right donuts, the dark red slice indicates the accumulative relative abundance of genera in the row-study which are also present in the column-study (averaged across samples). The grey slice indicates the accumulative relative abundance of the non-shared genera. Lower left donuts should be interpreted similarly, replacing column- and row-studies in the above description. In all 4 plots, only the healthy samples are considered.

Figure S2

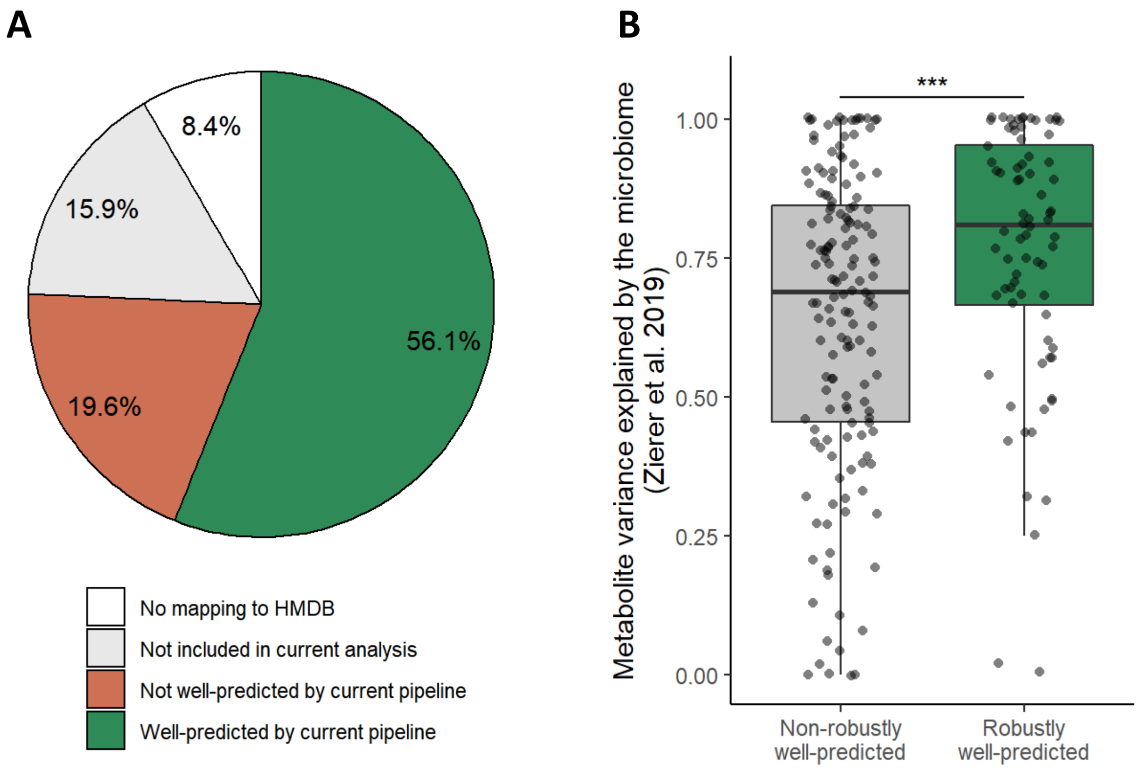

Figure S2: Comparisons to previous studies. (A) Comparison of the 107 metabolites identified as well-predicted by Mallick *et al.* (2019), using the FRANZOSA_IBD dataset, to predictability results obtained here on the same dataset. **(B)** Comparison of robustly well-predicted metabolites to estimates of the proportion of metabolite variance explained by the microbiome as calculated by Zierer *et al.* (2018). The association's significance was tested using a Mann-Whitney test. ***: $P < 0.001$.

Figure S3

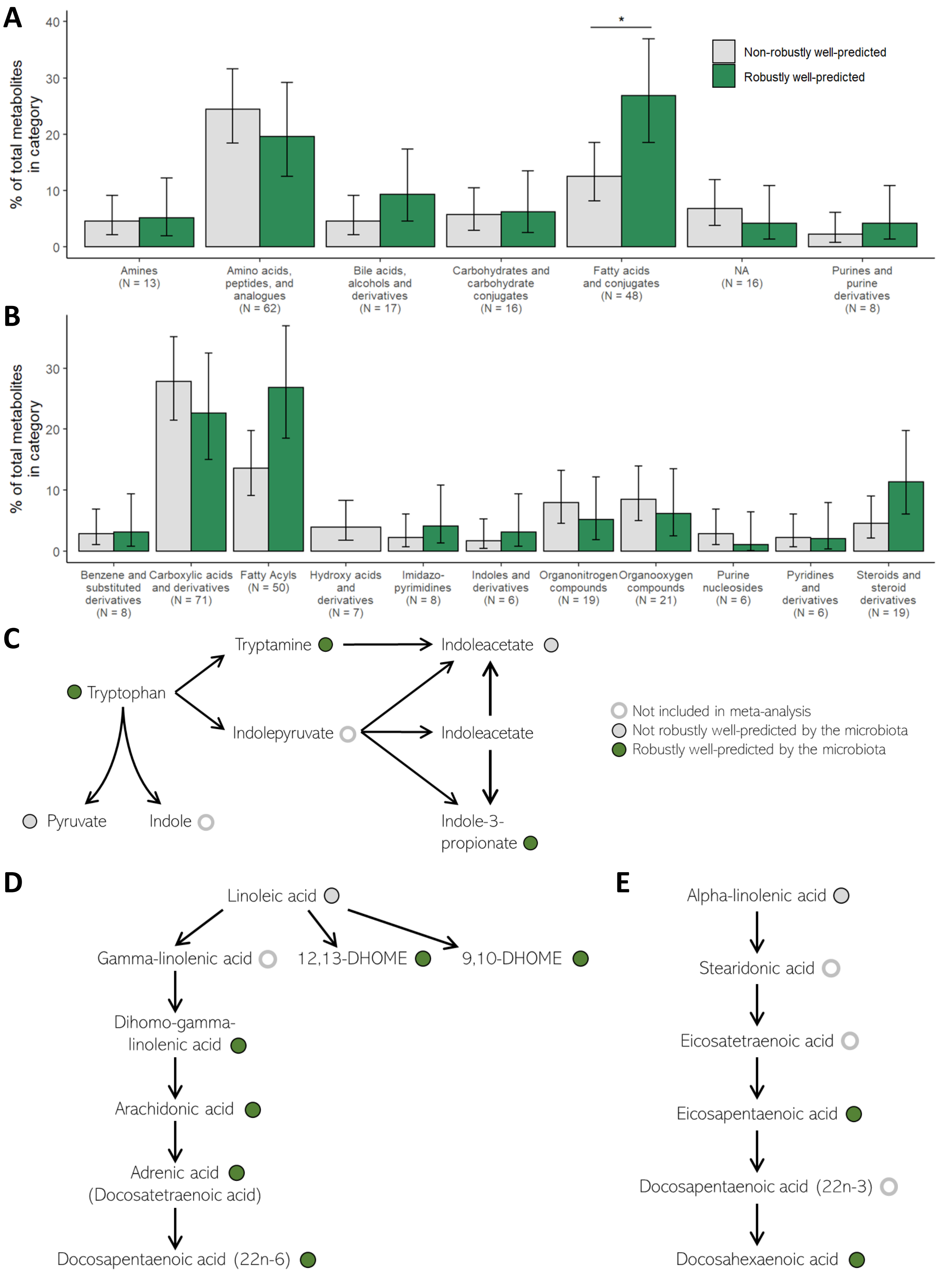

A
% of total metabolites in category
Non-robustly well-predicted
Robustly well-predicted
*
Amines (N = 13)
Amino acids, peptides, and analogues (N = 62)
Bile acids, alcohols and derivatives (N = 17)
Carbohydrates and carbohydrate conjugates (N = 16)
Fatty acids and conjugates (N = 48)
NA (N = 16)
Purines and purine derivatives (N = 8)
B
% of total metabolites in category
Benzene and substituted derivatives (N = 8)
Carboxylic acids and derivatives (N = 71)
Fatty Acyls (N = 50)
Hydroxy acids and derivatives (N = 7)
Imidazo-pyrimidines (N = 8)
Indoles and derivatives (N = 6)
Organonitrogen compounds (N = 19)
Organooxygen compounds (N = 21)
Purine nucleosides (N = 6)
Pyridines and derivatives (N = 6)
Steroids and steroid derivatives (N = 19)
C
Tryptophan
Tryptamine
Indoleacetate
Indolepyruvate
Indoleacetate
Pyruvate
Indole
Indole-3-propionate
Not included in meta-analysis
Not robustly well-predicted by the microbiota
Robustly well-predicted by the microbiota
D
Linoleic acid
Gamma-linolenic acid
12,13-DHOME
9,10-DHOME
Dihomo-gamma-linolenic acid
Arachidonic acid
Adrenic acid (Docosatetraenoic acid)
Docosapentaenoic acid (22n-6)
E
Alpha-linolenic acid
Stearidonic acid
Eicosatetraenoic acid
Eicosapentaenoic acid
Docosapentaenoic acid (22n-3)
Docosahexaenoic acid

Figure S3: Characteristics of robustly well-predicted metabolites. (A) Comparison of metabolite distribution over metabolite sub-classes in the HMDB's classification system. Classes with less than 6 metabolites were discarded from this plot. *: FDR-corrected P value < 0.05. **(B)** Comparison of metabolite distribution over metabolite classes in the HMDB's classification system. Classes with less than 6 metabolites were discarded. **(C)** An illustration of a part of the tryptophan metabolism pathway. Colored circles indicate whether the metabolite was robustly well-predicted by the gut microbiome (green), not robustly well-predicted (gray), or not included in the analysis (white). **(D)**, **(E)** An illustration of a part of the omega-3 and omega-6 metabolism pathways, respectively. Colored circles are as above.

Figure S4

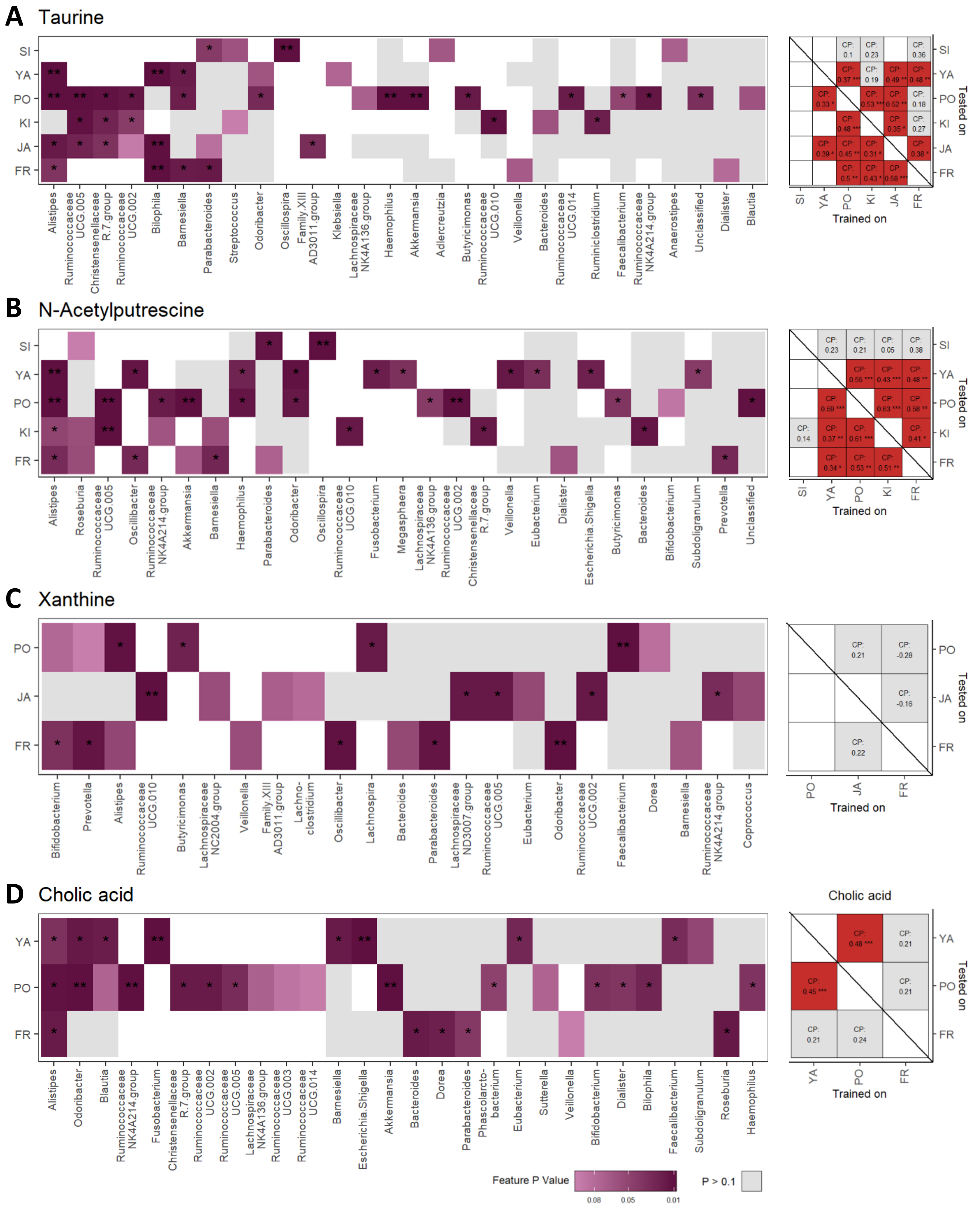
A Taurine
SI
YA
PO
KI
JA
FR
Alistipes
Ruminococcaceae UCG.005
Christensenellaceae R.7.group
Ruminococcaceae UCG.002
Bilophila
Barnesiella
Parabacteroides
Streptococcus
Odoribacter
Oscillospira
Family.XIII AD3011.group
Klebsiella
Lachnospiraceae NK4A136.group
Haemophilus
Akkermansia
Adlercreutzia
Butyricimonas
Ruminococcaceae UCG.010
Veillonella
Bacteroides
Ruminococcaceae UCG.014
Ruminiclostridium
Faecalibacterium
Ruminococcaceae NK4A214.group
Anaerostipes
Unclassified
Dialister
Blautia
Trained on
Tested on
B N-Acetylputrescine
Alistipes
Roseburia
Ruminococcaceae UCG.005
Oscillibacter
Ruminococcaceae NK4A214.group
Akkermansia
Barnesiella
Haemophilus
Parabacteroides
Odoribacter
Oscillospira
Ruminococcaceae UCG.010
Fusobacterium
Megasphaera
Lachnospiraceae NK4A136.group
Ruminococcaceae UCG.002
Christensenellaceae R.7.group
Veillonella
Eubacterium
Dialister
Escherichia.Shigella
Butyricimonas
Bacteroides
Bifidobacterium
Subdoligranulum
Prevotella
Unclassified
Trained on
Tested on
C Xanthine
Bifidobacterium
Prevotella
Alistipes
Ruminococcaceae UCG.010
Butyricimonas
Lachnospiraceae NC2004.group
Veillonella
Family.XIII AD3011.group
Lachno-clostridium
Oscillibacter
Lachnospira
Bacteroides
Parabacteroides
Lachnospiraceae ND3007.group
Ruminococcaceae UCG.005
Eubacterium
Odoribacter
Ruminococcaceae UCG.002
Faecalibacterium
Dorea
Barnesiella
Ruminococcaceae NK4A214.group
Coprococcus
Trained on
Tested on
D Cholic acid
Alistipes
Odoribacter
Blautia
Ruminococcaceae NK4A214.group
Fusobacterium
Christensenellaceae R.7.group
Ruminococcaceae UCG.002
Ruminococcaceae UCG.005
Lachnospiraceae NK4A136.group
Ruminococcaceae UCG.003
Ruminococcaceae UCG.014
Barnesiella
Escherichia.Shigella
Akkermansia
Bacteroides
Dorea
Parabacteroides
Phascolarcto-bacterium
Eubacterium
Sutterella
Veillonella
Bifidobacterium
Dialister
Bilophila
Faecalibacterium
Subdoligranulum
Roseburia
Haemophilus
Cholic acid
Trained on
Tested on
Feature P Value
0.08
0.05
0.01
P > 0.1

Figure S4: Comparisons of metabolite models between datasets. Detailed model comparison for taurine **(A)**, N-acetylputrescine **(B)**, xanthine **(C)** and cholic acid **(D)** metabolites. Panel explanations are as described in Figure 4.

Figure S5

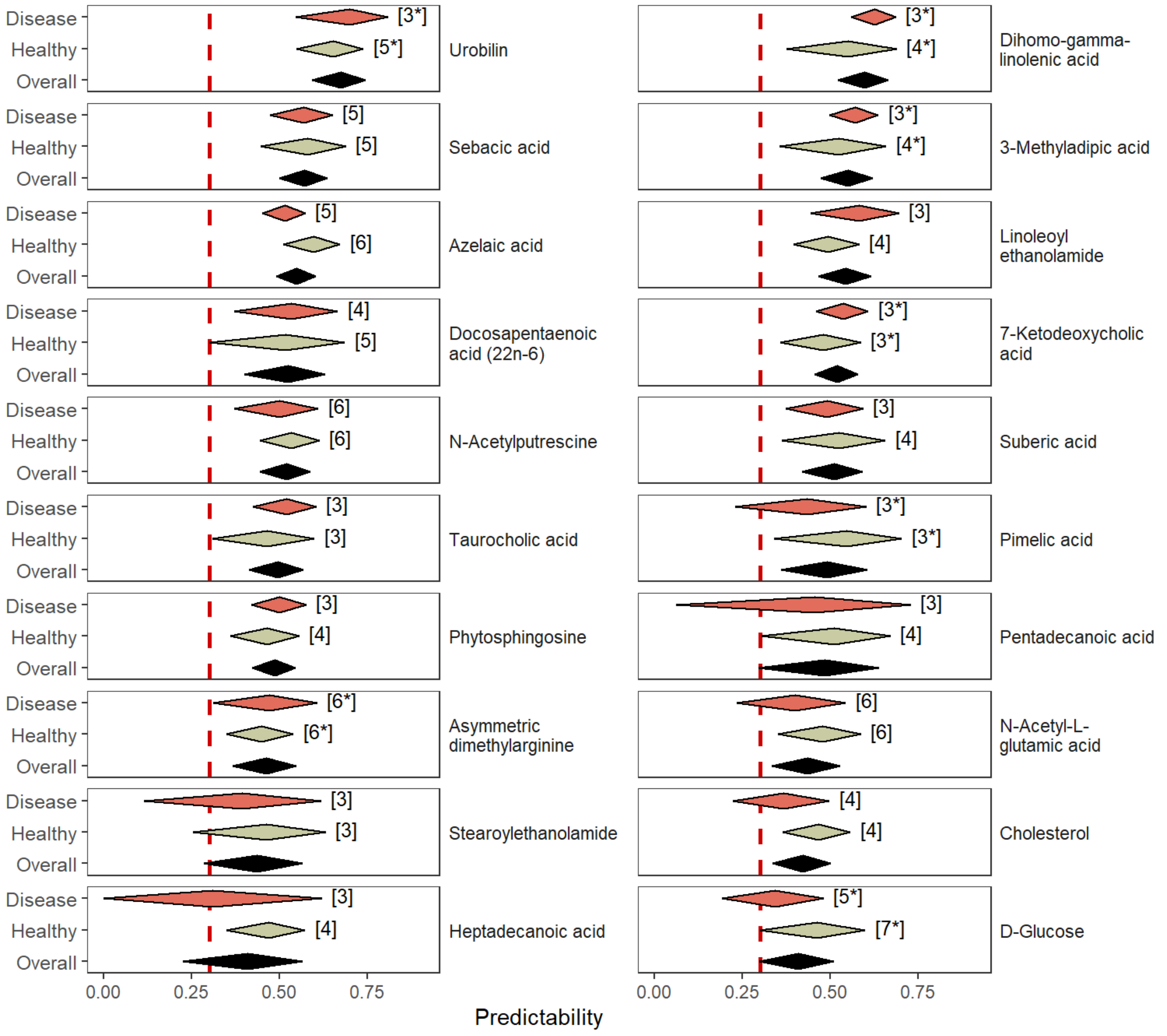

Figure S5: Examples of disease-independent robustly well-predicted metabolites. Top 20 robustly well-predicted metabolites which were also robustly well-predicted in disease. Diamonds and adjacent labels represent REM estimated mean predictability and the number of datasets, respectively, with a star indicating that this metabolite was annotated with low confidence for one or more studies. The red dashed line represents a Spearman's correlation of 0.3, which we defined as the threshold for a "well-performing" predictive model. The 3 diamonds represent the estimated effects of disease datasets, healthy datasets, and all datasets, respectively.

Figure S6

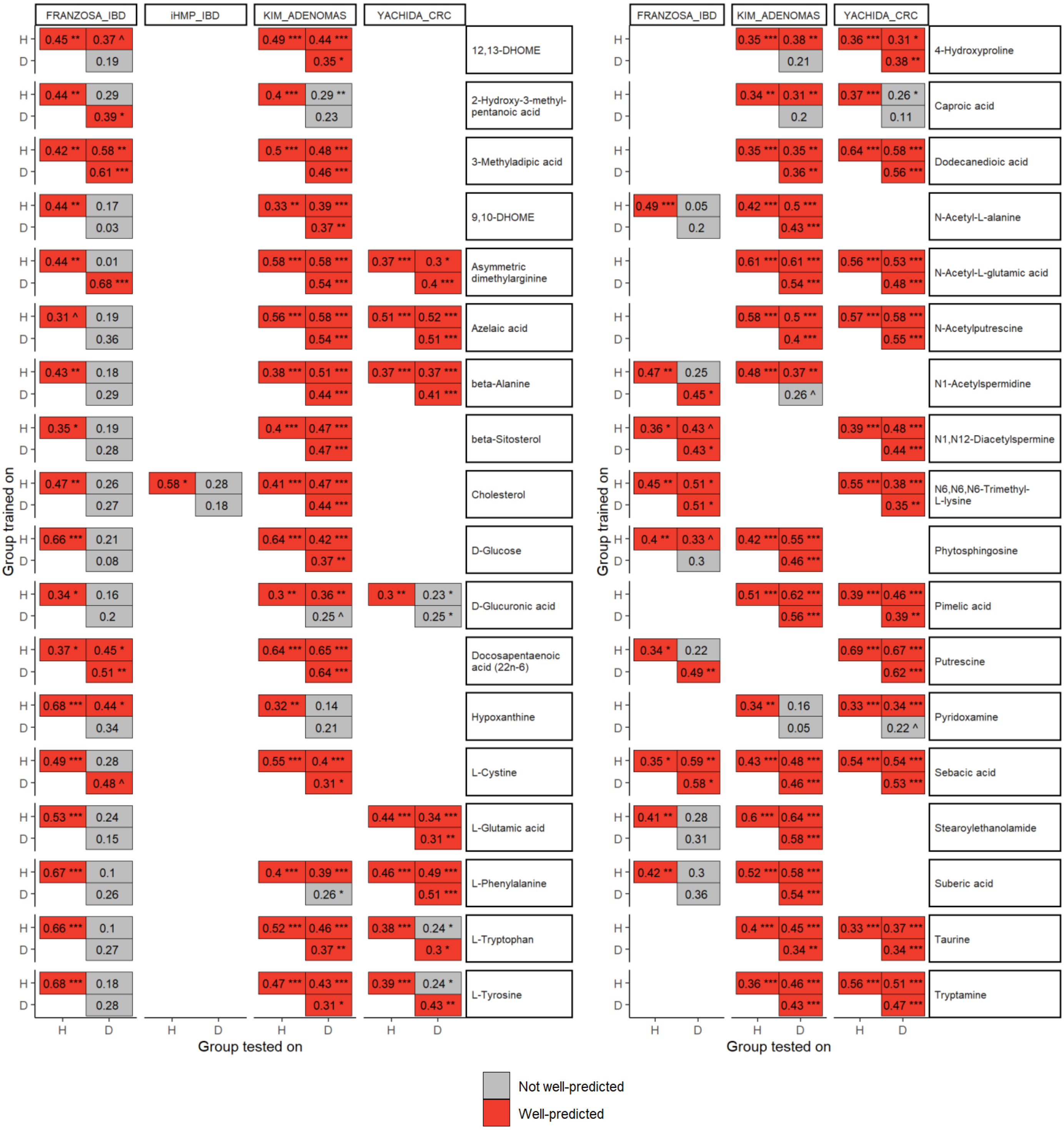
FRANZOSA_IBD
iHMP_IBD
KIM_ADENOMAS
YACHIDA_CRC
12,13-DHOME
0.45 ** 0.37 ^ 0.49 *** 0.44 ***
0.19 0.35 *
2-Hydroxy-3-methyl-pentanoic acid
0.44 ** 0.29 0.4 *** 0.29 **
0.39 * 0.23
3-Methyladipic acid
0.42 ** 0.58 ** 0.5 *** 0.48 ***
0.61 *** 0.46 ***
9,10-DHOME
0.44 ** 0.17 0.33 ** 0.39 ***
0.03 0.37 **
Asymmetric dimethylarginine
0.44 ** 0.01 0.58 *** 0.58 *** 0.37 *** 0.3 *
0.68 *** 0.54 *** 0.4 ***
Azelaic acid
0.31 ^ 0.19 0.56 *** 0.58 *** 0.51 *** 0.52 ***
0.36 0.54 *** 0.51 ***
beta-Alanine
0.43 ** 0.18 0.38 *** 0.51 *** 0.37 *** 0.37 ***
0.29 0.44 *** 0.41 ***
beta-Sitosterol
0.35 * 0.19 0.4 *** 0.47 ***
0.28 0.47 ***
Cholesterol
0.47 ** 0.26 0.58 * 0.28 0.41 *** 0.47 ***
0.27 0.18 0.44 ***
D-Glucose
0.66 *** 0.21 0.64 *** 0.42 ***
0.08 0.37 **
D-Glucuronic acid
0.34 * 0.16 0.3 ** 0.36 ** 0.3 ** 0.23 *
0.2 0.25 ^ 0.25 *
Docosapentaenoic acid (22n-6)
0.37 * 0.45 * 0.64 *** 0.65 ***
0.51 ** 0.64 ***
Hypoxanthine
0.68 *** 0.44 * 0.32 ** 0.14
0.34 0.21
L-Cystine
0.49 *** 0.28 0.55 *** 0.4 ***
0.48 ^ 0.31 *
L-Glutamic acid
0.53 *** 0.24 0.44 *** 0.34 ***
0.15 0.31 **
L-Phenylalanine
0.67 *** 0.1 0.4 *** 0.39 *** 0.46 *** 0.49 ***
0.26 0.26 * 0.51 ***
L-Tryptophan
0.66 *** 0.1 0.52 *** 0.46 *** 0.38 *** 0.24 *
0.27 0.37 ** 0.3 *
L-Tyrosine
0.68 *** 0.18 0.47 *** 0.43 *** 0.39 *** 0.24 *
0.28 0.31 * 0.43 **
Group trained on
H
D
Group tested on
FRANZOSA_IBD
KIM_ADENOMAS
YACHIDA_CRC
4-Hydroxyproline
0.35 *** 0.38 ** 0.36 *** 0.31 *
0.21 0.38 **
Caproic acid
0.34 ** 0.31 ** 0.37 *** 0.26 *
0.2 0.11
Dodecanedioic acid
0.35 *** 0.35 ** 0.64 *** 0.58 ***
0.36 ** 0.56 ***
N-Acetyl-L-alanine
0.49 *** 0.05 0.42 *** 0.5 ***
0.2 0.43 ***
N-Acetyl-L-glutamic acid
0.61 *** 0.61 *** 0.56 *** 0.53 ***
0.54 *** 0.48 ***
N-Acetylputrescine
0.58 *** 0.5 *** 0.57 *** 0.58 ***
0.4 *** 0.55 ***
N1-Acetylspermidine
0.47 ** 0.25 0.48 *** 0.37 **
0.45 * 0.26 ^
N1,N12-Diacetylspermine
0.36 * 0.43 ^ 0.39 *** 0.48 ***
0.43 * 0.44 ***
N6,N6,N6-Trimethyl-L-lysine
0.45 ** 0.51 * 0.55 *** 0.38 ***
0.51 * 0.35 **
Phytosphingosine
0.4 ** 0.33 ^ 0.42 *** 0.55 ***
0.3 0.46 ***
Pimelic acid
0.51 *** 0.62 *** 0.39 *** 0.46 ***
0.56 *** 0.39 **
Putrescine
0.34 * 0.22 0.69 *** 0.67 ***
0.49 ** 0.62 ***
Pyridoxamine
0.34 ** 0.16 0.33 *** 0.34 ***
0.05 0.22 ^
Sebacic acid
0.35 * 0.59 ** 0.43 *** 0.48 *** 0.54 *** 0.54 ***
0.58 * 0.46 *** 0.53 ***
Stearoylethanolamide
0.41 ** 0.28 0.6 *** 0.64 ***
0.31 0.58 ***
Suberic acid
0.42 ** 0.3 0.52 *** 0.58 ***
0.36 0.54 ***
Taurine
0.4 *** 0.45 *** 0.33 *** 0.37 ***
0.34 ** 0.34 ***
Tryptamine
0.36 *** 0.46 *** 0.56 *** 0.51 ***
0.43 *** 0.47 ***
Group trained on
Group tested on
Not well-predicted
Well-predicted

Figure S6: Transferability of metabolite models from control to cases within the same study. Cross-predictability analysis results are presented for each case-control study and each metabolite analyzed (see Methods). Only metabolites analyzed in at least 2 datasets are presented. In each panel, the upper left box represents model performance when trained and tested on the control group, the lower right box represents model performance when trained and tested on the cases group, and the upper right box represents model performance when trained on controls and tested on cases. Numbers in cells indicate Spearman's correlation between predicted and actual metabolite levels. Red cells indicate that the metabolite model was considered well-predicted. ^: $P < 0.1$; *: $P < 0.05$; **: $P < 0.01$; ***: $P < 0.001$.

Figure S7

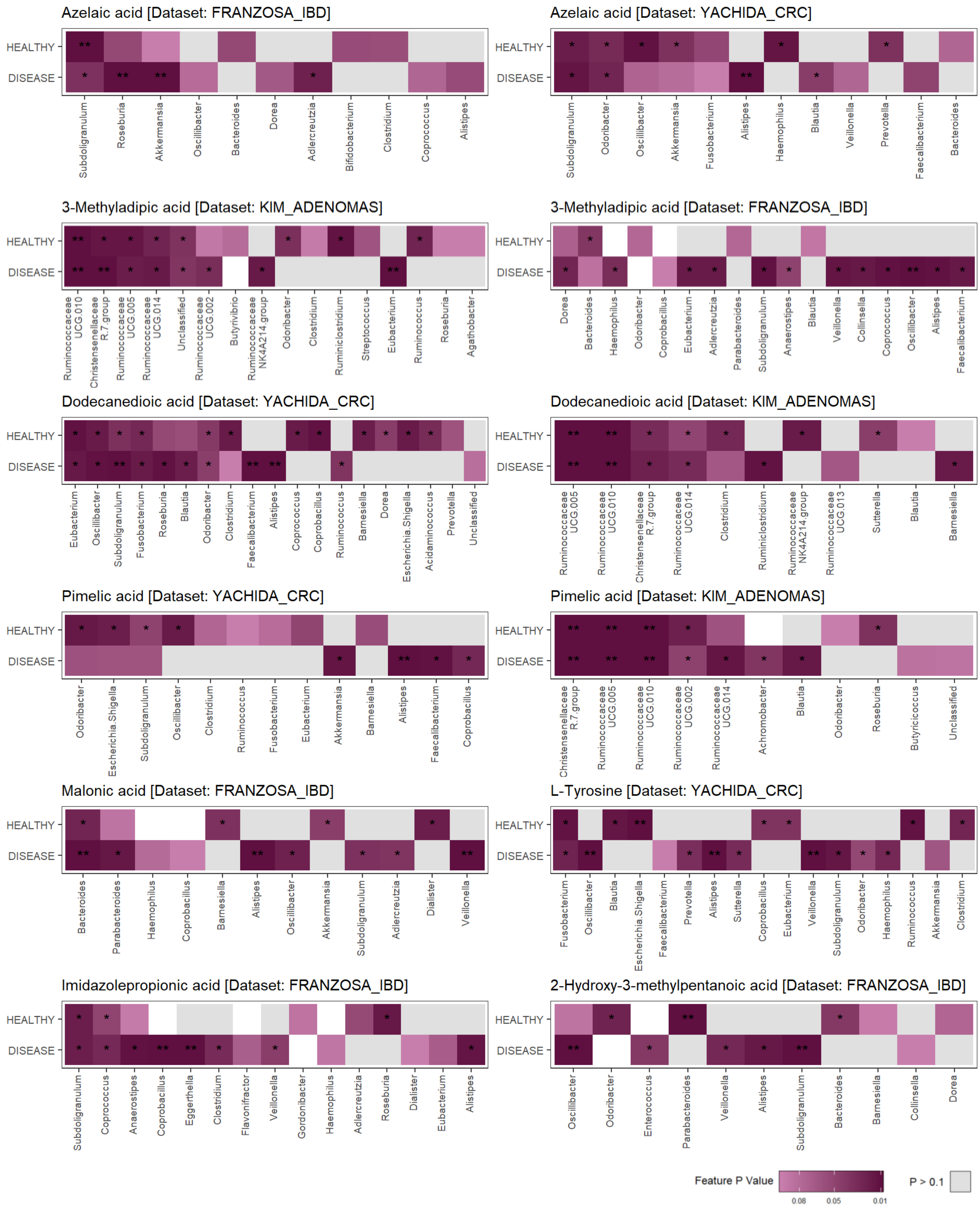

Azelaic acid [Dataset: FRANZOSA_IBD]
HEALTHY
DISEASE
Subdoligranulum Roseburia Akkermansia Oscillibacter Bacteroides Dorea Adlercreutzia Bifidobacterium Clostridium Coprococcus Alistipes
Azelaic acid [Dataset: YACHIDA_CRC]
Subdoligranulum Odoribacter Oscillibacter Akkermansia Fusobacterium Alistipes Haemophilus Blautia Veillonella Prevotella Faecalibacterium Bacteroides
3-Methyladipic acid [Dataset: KIM_ADENOMAS]
Ruminococcaceae UCG.010 Christensenellaceae R.7.group Ruminococcaceae UCG.005 Ruminococcaceae UCG.014 Unclassified Ruminococcaceae UCG.002 Butyrivibrio Ruminococcaceae NK4A214.group Odoribacter Clostridium Ruminiclostridium Streptococcus Eubacterium Ruminococcus Roseburia Agathobacter
3-Methyladipic acid [Dataset: FRANZOSA_IBD]
Dorea Bacteroides Haemophilus Odoribacter Coprobacillus Eubacterium Adlercreutzia Parabacteroides Subdoligranulum Anaerostipes Blautia Veillonella Collinsella Coprococcus Oscillibacter Alistipes Faecalibacterium
Dodecanedioic acid [Dataset: YACHIDA_CRC]
Eubacterium Oscillibacter Subdoligranulum Fusobacterium Roseburia Blautia Odoribacter Clostridium Faecalibacterium Alistipes Coprococcus Coprobacillus Ruminococcus Barnesiella Dorea Escherichia.Shigella Acidaminococcus Prevotella Unclassified
Dodecanedioic acid [Dataset: KIM_ADENOMAS]
Ruminococcaceae UCG.005 Ruminococcaceae UCG.010 Christensenellaceae R.7.group Ruminococcaceae UCG.014 Clostridium Ruminiclostridium Ruminococcaceae NK4A214.group Ruminococcaceae UCG.013 Sutterella Blautia Barnesiella
Pimelic acid [Dataset: YACHIDA_CRC]
Odoribacter Escherichia.Shigella Subdoligranulum Oscillibacter Clostridium Ruminococcus Fusobacterium Eubacterium Akkermansia Barnesiella Alistipes Faecalibacterium Coprobacillus
Pimelic acid [Dataset: KIM_ADENOMAS]
Christensenellaceae R.7.group Ruminococcaceae UCG.005 Ruminococcaceae UCG.010 Ruminococcaceae UCG.002 Ruminococcaceae UCG.014 Achromobacter Blautia Odoribacter Roseburia Butyricicoccus Unclassified
Malonic acid [Dataset: FRANZOSA_IBD]
Bacteroides Parabacteroides Haemophilus Coprobacillus Barnesiella Alistipes Oscillibacter Akkermansia Subdoligranulum Adlercreutzia Dialister Veillonella
L-Tyrosine [Dataset: YACHIDA_CRC]
Fusobacterium Oscillibacter Blautia Escherichia.Shigella Faecalibacterium Prevotella Alistipes Sutterella Coprobacillus Eubacterium Veillonella Subdoligranulum Odoribacter Haemophilus Ruminococcus Akkermansia Clostridium
Imidazolepropionic acid [Dataset: FRANZOSA_IBD]
Subdoligranulum Coprococcus Anaerostipes Coprobacillus Eggerthella Clostridium Flavonifractor Veillonella Gordonibacter Haemophilus Adlercreutzia Roseburia Dialister Eubacterium Alistipes
2-Hydroxy-3-methylpentanoic acid [Dataset: FRANZOSA_IBD]
Oscillibacter Odoribacter Enterococcus Parabacteroides Veillonella Alistipes Subdoligranulum Bacteroides Barnesiella Collinsella Dorea
Feature P Value
0.08 0.05 0.01
P > 0.1

Figure S7: Examples of genus significant contributors' comparison between healthy and disease metabolite models. Each panel describes significant contributors' comparison for a specific metabolite and dataset. Here upper rows and lower rows represent the control group and case group, respectively, from the same study. Colors and significance marks are as described in Figure 4.

Figure S8

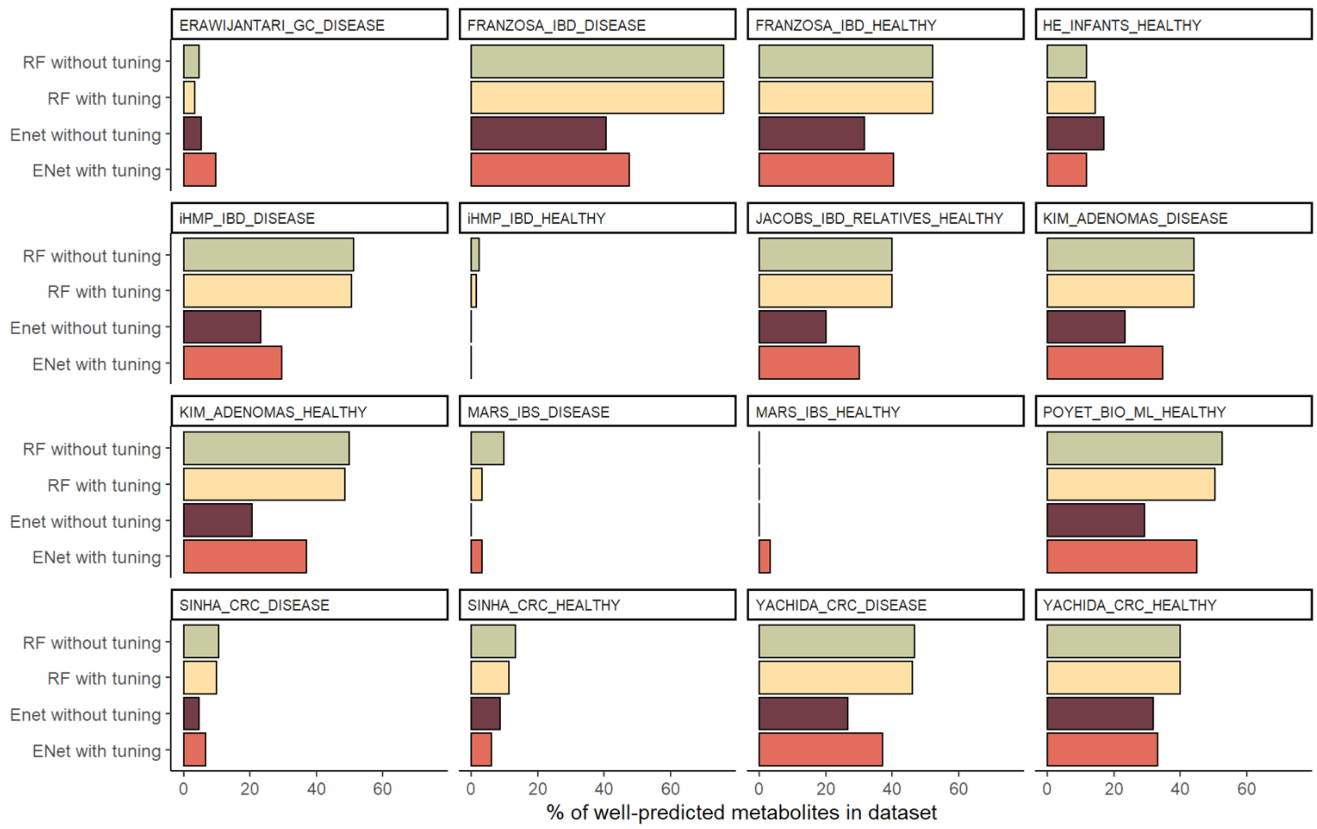

Figure S8: Comparison of different machine learning pipelines (related to supplementary note 1). Each panel describes the percent of well-predicted metabolites out of all metabolites analyzed generated by each machine learning pipeline, in a specific dataset. Both healthy and disease datasets are presented.
